# Accelerated Evolution of the *Prdm9* Speciation Gene across Diverse Metazoan Taxa

**DOI:** 10.1371/journal.pgen.1000753

**Published:** 2009-12-04

**Authors:** Peter L. Oliver, Leo Goodstadt, Joshua J. Bayes, Zoë Birtle, Kevin C. Roach, Nitin Phadnis, Scott A. Beatson, Gerton Lunter, Harmit S. Malik, Chris P. Ponting

**Affiliations:** 1 Medical Research Council Functional Genomics Unit, Department of Physiology, Anatomy and Genetics, University of Oxford, Oxford, United Kingdom; 2 Department of Molecular and Cell Biology, University of California Berkeley, Berkeley, California, United States of America; 3 Division of Basic Sciences, Fred Hutchinson Cancer Research Center, Seattle, Washington, United States of America; 4 Department of Genome Sciences, University of Washington, Seattle, Washington, United States of America; 5 School of Chemistry and Molecular Biosciences, University of Queensland, Brisbane, Queensland, Australia; 6 Howard Hughes Medical Institute, Fred Hutchinson Cancer Research Center, Seattle, Washington, United States of America; University of Arizona, United States of America

## Abstract

The onset of prezygotic and postzygotic barriers to gene flow between populations is a hallmark of speciation. One of the earliest postzygotic isolating barriers to arise between incipient species is the sterility of the heterogametic sex in interspecies' hybrids. Four genes that underlie hybrid sterility have been identified in animals: *Odysseus*, *JYalpha*, and *Overdrive* in *Drosophila* and *Prdm9* (*Meisetz*) in mice. Mouse *Prdm9* encodes a protein with a KRAB motif, a histone methyltransferase domain and several zinc fingers. The difference of a single zinc finger distinguishes *Prdm9* alleles that cause hybrid sterility from those that do not. We find that concerted evolution and positive selection have rapidly altered the number and sequence of *Prdm9* zinc fingers across 13 rodent genomes. The patterns of positive selection in *Prdm9* zinc fingers imply that rapid evolution has acted on the interface between the Prdm9 protein and the DNA sequences to which it binds. Similar patterns are apparent for Prdm9 zinc fingers for diverse metazoans, including primates. Indeed, allelic variation at the DNA–binding positions of human PRDM9 zinc fingers show significant association with decreased risk of infertility. *Prdm9* thus plays a role in determining male sterility both between species (mouse) and within species (human). The recurrent episodes of positive selection acting on *Prdm9* suggest that the DNA sequences to which it binds must also be evolving rapidly. Our findings do not identify the nature of the underlying DNA sequences, but argue against the proposed role of *Prdm9* as an essential transcription factor in mouse meiosis. We propose a hypothetical model in which incompatibilities between Prdm9-binding specificity and satellite DNAs provide the molecular basis for *Prdm9*-mediated hybrid sterility. We suggest that *Prdm9* should be investigated as a candidate gene in other instances of hybrid sterility in metazoans.

## Author Summary

Speciation, the process by which one species splits into two, involves reproductive barriers between previously interbreeding populations. The question of how speciation occurs has rightly occupied the attention of biologists since before Darwin's “On the Origin of Species.” Studies of recently diverged species have revealed the presence of hybrid sterility genes (colloquially referred to as “speciation genes”), alleles of which are associated with sterility of interspecies hybrids. Mouse *Prdm9* is the only known such gene in vertebrate animals. Here we report that the Prdm9 protein has evolved extremely rapidly in its DNA-binding domain, comprising an array of “zinc fingers.” This suggests that hybrid sterility may arise from a mismatch between the DNA-binding specificity of Prdm9 and rapidly evolving DNA. We propose that Prdm9 binds to satellite-DNA repeats evolving rapidly within and between different species. Prdm9 evolution is unusual because other hybrid sterility genes appear only to evolve rapidly in isolated bursts, whereas Prdm9 has evolved rapidly over 700 million years, in many rodent species, diverse primates and other metazoans. This leads to the tantalizing possibility that Prdm9 may have served as a “speciation gene” on other occasions in metazoan evolution, a possibility that will now need to be investigated.

## Introduction

The question of how two species originate from one has fascinated biologists since before Darwin's iconic treatise on the subject [1]. Postzygotic reproductive barriers between species are thought to result from the acquisition of genetic incompatibilities as an incidental by-product of divergence between two populations. In its simplest form, this Dobzhansky-Muller model involves genetic interactions between two loci (e.g. *a* and *b*) [2]. In isolated populations, new alleles can arise and go to fixation in two isolated populations (*A* in one and *B* in the other) since they remain compatible with ancestral alleles. However, a negative epistatic interaction between the two new alleles (*A* with *B*) in hybrids might result in sterility or inviability, a hallmark of postzygotic isolation in hybrids between two species [3]. Theory predicts that additional incompatibilities will accumulate rapidly following an initial genetic incompatibility [4]. One of the earliest postzygotic isolating barriers in interspecies hybrids is the sterility of the heterogametic sex (XY males or ZW females), a pattern referred to as Haldane's rule that holds almost universally across animal taxa [3],[5]. Examination of early events in speciation that lead to hybrid sterility (for example [6],[7]) is thus vital to gain insight into this mysterious process.

The first hybrid sterility gene to be discovered was the *Drosophila Odysseus-site homeobox* (*OdsH*) gene. The *D. mauritiana* allele of *OdsH* causes hybrid male sterility when introgressed into *D. simulans* together with adjacent loci [8],[9]. *OdsH* encodes a presumptive DNA-binding protein which is exclusively expressed in male reproductive tissues [9]. *OdsH* function within *Drosophila* species remained unclear until recently (ablation of the gene in *D. melanogaster* has a very modest effect on male fertility [10]) as did the molecular basis for why it causes hybrid sterility. However, the manifestation of hybrid sterility appears to be correlated with rapid evolution of *OdsH* specifically in its DNA-binding homeobox domain, in the species clade that includes *D. mauritiana* and *D. simulans* [11].

A second hybrid sterility gene was discovered not as a Dobzhansky-Muller incompatibility but as a result of gene transposition. Hybrids between *D. melanogaster* and *D. simulans*, which carry two 4^th^ chromosomes from *D. simulans* in an otherwise *D. melanogaster* genetic background, are sterile. This sterility is caused by the transposition of the *JYAlpha* gene away from the 4^th^ chromosome in *D. simulans* [12]. Since *JYAlpha* is required for male fertility, *D. melanogaster* male flies that only possess *D. simulans* 4^th^ chromosomes lack *JYAlpha* and are therefore sterile. In contrast to *OdsH*, the biological cause of hybrid sterility is well understood but involves no sequence divergence of the underlying sterility gene and only affects a fraction of F2 hybrids.

A third hybrid sterility gene was recently discovered in crosses between the Bogota and USA subpopulations of *D. pseudoobscura*. F1 males resulting from crosses between Bogota females and USA males are almost completely sterile when young. When aged, however, these F1 males recover partial fertility but produce all female progeny. Intriguingly, a single gene *Overdrive (Ovd)* was found to be causal for both the segregation distortion and hybrid male sterility [13]. Like *OdsH*, *Ovd* encodes a putative DNA-binding protein whose biological function is unclear. Like *OdsH*, rapid evolution of *Ovd* in the Bogota lineage appears to be associated with hybrid sterility. Genetic results with *Ovd* strongly suggest that hybrid sterility is a by-product of intraspecies genomic conflict, manifest as segregation distortion [13].

**Figure 1:**
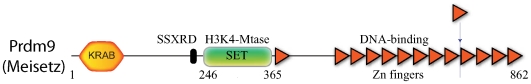
Schematic of Prdm9 protein encoded by *M. musculus*. Schematic of the domain architecture for the long protein isoform encoded by the *M. musculus Prdm9* gene. The Prdm9 protein contains KRAB, SSXRD, and SET domains and a single zinc finger in its N-terminal half, while the C-terminal half consists of an array of zinc finger domains [17]. The shorter Prdm9 protein isoforms lack the C-terminal zinc fingers and apparently do not localize to the nucleus. Sterility and fertility associated alleles of *Prdm9* in *M. m. musculus* differ only in one extra zinc finger (red triangle) [15].

*Prdm9 (Meisetz)* is the fourth hybrid sterility gene, the first to be described in vertebrates. It was discovered in crosses between the mouse subspecies *Mus musculus musculus* and *Mus musculus domesticus*. Allelic differences at *Prdm9* provide the genetic basis for the *Hybrid sterility 1* (*Hst1*) locus, which together with other genetic loci [6],[7],[14], is responsible for spermatogenic failure in sterile hybrids between *Mus m. musculus* and *Mus m. domesticus* [15]. Polymorphism linked to *Hst1* is associated with sterility traits not only for *Mus m. domesticus* strains but also, separately, for *Mus m. musculus* strains [16]. In natural *Mus m. musculus* populations these polymorphisms appear to have arisen very recently [16]. *Prdm9* is a meiosis-specific gene that is only expressed in germ cells entering meiotic prophase in both female and male mice [17]. Loss of *Prdm9* causes sterility in both sexes due to impaired meiotic progression at the pachytene stage. Furthermore, nonsynonymous SNPs in human *PRDM9* are associated with infertility and azoospermia via meiotic arrest [18],[19]. *Prdm9* encodes 3 protein isoforms, of which the largest isoform contains an N-terminal KRAB motif, a central histone H3 Lysine-4-methyltransferase (SET) domain, and several zinc fingers in its carboxy-terminal region (Figure 1). Similar zinc fingers in other proteins have been shown to mediate sequence-specific binding to DNA. The number of zinc fingers encoded in mouse *Prdm9* appears to directly affect hybrid sterility. Whereas an allele of *Prdm9* encoding 13 zinc fingers causes postzygotic hybrid sterility, an allele containing 14 zinc fingers does not (Figure 1) [15]. The finding that changes in a single DNA-binding determinant appears to be causal for hybrid sterility motivated our analysis to study the evolutionary constraints that shape the sequence and copy number of zinc finger motifs in *Prdm9* across a broad taxonomic panel of metazoans, starting with rodents.

## Results

### Concerted evolution and positive selection of *Prdm9*-encoded zinc fingers in rodents

**Figure 2:**
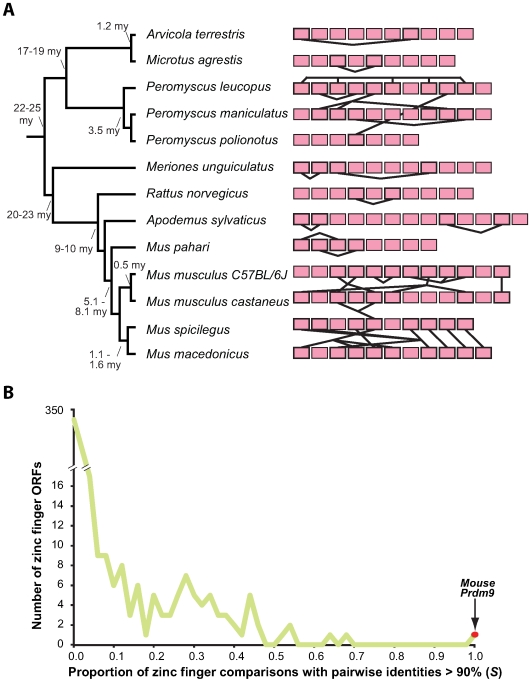
Concerted evolution among rodent *Prdm9* genes. (A) *Prdm9* C-terminal zinc fingers for 13 rodent species are shown as pink rectangles. Zinc fingers whose nucleotide sequences are identical are joined by solid lines. Zinc fingers with identical sequences from the same species are consistent with gene conversion and/or intra-exon duplication. A phylogeny of these species is also shown with estimated divergence dates (indicated at nodes) given in millions of years (my) [20],[83]. Common names to species are listed in the legend to Figure 6. (B) The proportion of pairwise cDNA comparisons between aligned zinc fingers from the same gene (see Materials and Methods) which show greater than 90% identity. All mouse *Prdm9* zinc fingers are more than 90% identical to all other C-terminal zinc fingers in the same protein (indicated in red), a much higher fraction than for any other zinc finger protein encoded by the mouse genome.

We sequenced the terminal zinc fingers from the final exons of *Prdm9* from 11 rodent species to which we added the genomic sequences of mouse (C57BL/6J) and rat *Prdm9* (Figure 2A), thereby sampling a ∼25 million year period of rodent phylogeny [20]. The C57BL/6J strain of mice is a mosaic of *M.m. musculus*, *M.m. domesticus* and *M.m. castaneus* [21]. The C57BL/6J mouse genome assembly harbours the *M.m. domesticus Prdm9* allele [22]. We found that rodents vary greatly in their numbers of zinc fingers present in the C-terminal array: from 7 in *Peromyscus polionotus* to 12 in *Mus musculus* (Figure 2A). Even closely-related species pairs, such as field and water voles (*Microtus agrestis* and *Arvicola terrestris*), and *M. macedonicus* and *M. spicilegus*, differ in their numbers of zinc fingers (Figure 2A).

Rodent *Prdm9* zinc finger sequences have been subject to concerted evolution. Many changes in numbers of zinc fingers have resulted from very recent lineage-specific duplications (Figure 2A). Twelve of the 13 rodent species we examined possess at least one pair of *Prdm9* zinc fingers that were so recently duplicated that they have identical nucleotide sequences. In one case (*Peromyscus leucopus*, Figure 2A), *Prdm9* encodes a cluster of five zinc fingers that are identical at the nucleotide level, together with another pair of identical zinc fingers. Consistent with concerted evolution, *Prdm9* zinc fingers from the same species often form monophyletic clades, even in comparisons of closely related rodents (Figure S1). Such concerted sequence evolution may result from multiple rounds of zinc finger duplication and deletion (‘birth-and-death’ model [23]) to change zinc finger numbers. However, we favor non-allelic gene conversion as a dominant mechanism [24] since it more easily accounts for the many interdigitated and non-adjacent zinc finger duplications, as well as the complexity of the inferred zinc finger phylogeny. Although more occasional gain and loss of zinc finger sequences have been observed previously for other genes [25], the extreme degree of sequence similarity between different zinc finger pairs is far greater for *Prdm9* than for any other zinc finger gene present in the C57BL/6J mouse genome sequence (Figure 2B).

**Figure 3:**
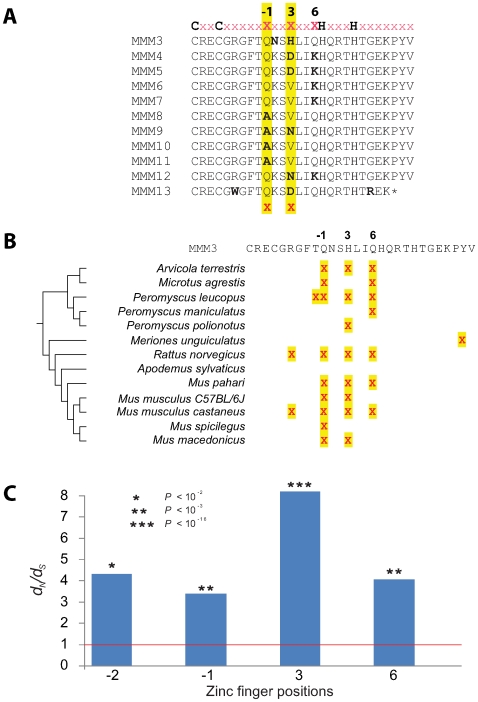
Positive selection of zinc fingers encoded by rodent *Prdm9* genes. (A) A multiple alignment of the zinc finger sequences from *M. musculus* C57BL/6J highlights the invariant Cys_2_His_2_ Zn^2+^-coordinating residues as well as positions −1, 3, and 6 that dictate the DNA-binding specificity of individual zinc fingers. Deviations from the consensus amino acid at each position are shown in boldface. In this species, positions −1 and 3 meet the criteria for positive selection [27] (highlighted in yellow and with red crosses). (B) Predicted positively selected sites in *Prdm9* from diverse rodent lineages. Positive selection was inferred for each species [27] from intra-species *Prdm9* zinc finger sequence alignments. Positively selected sites (*P*<0.05 after multiple testing correction) are shown mapped to the third mouse Prdm9 zinc finger sequence (MMM3). The majority of positively selected sites fall at positions −1, 3, and 6. (C) Estimated *d_N_/d_S_* values at four zinc finger positions (namely, −2, −1, 3, and 6) in a comparison of zinc fingers from all rodents (in contrast to the analyses of species-specific zinc fingers in (B)) for which there exists strong evidence of positive selection [27]. The *P*-values shown have been corrected for multiple testing. Common names to species are listed in the legend to Figure 6.

In addition to concerted evolution, our analyses reveal evidence for positive selection at particular codons responsible for DNA binding specificities within *Prdm9* zinc fingers in rodents. Due to the high degree of concerted evolution, it is not formally correct to carry out a pairwise analysis of the non-synonymous to synonymous rate ratio (*d_N_/d_S_*) when comparing *Prdm9* sequences from two different species. Instead, by comparing all *Prdm9* zinc fingers within a species, we find that all but one of these 13 rodent species have acquired more amino acid substitutions than would be expected under neutral evolution within their *Prdm9* zinc fingers (Figure 3). For instance, in the *Prdm9* encoded zinc fingers from *Mus musculus* strain C57BL/6J (Figure 3A), two codons are predicted to have evolved under positive selection (positions labelled −1 and 3 in Figure 3A). Intriguingly, positive selection is restricted to only a small number of positions within these zinc finger sequences. Sites labelled −1, 3, and 6 were identified as having evolved by positive selection in the majority of the 13 rodent species we examined when comparing all zinc fingers from a particular species (tabulated in Figure 3B). Codons at these sites are turned over rapidly. For instance, two recently diverged vole species, *Microtus agrestis* and *Arvicola terrestris* exhibit species-specific codons at positions −1, 3 and 6 (Figure S2) despite their independent evolution only over the last 0.5 million years [26]. In each case, we use the Sitewise Likelihood-ratio method (SLR) [27] with *p-*value thresholds of 0.05 after multiple testing correction. Since these methods can be strongly affected by tree topology, we tested both the most likely and other competing topologies to conservatively estimate non-synonymous substitutions; this will reduce the chance of false-positives in our analysis (see Materials and Methods). These unusually elevated values may reflect the sustained action of positive selection, consistent with the elevated rates observed for many rodent species (Figure 3). Rapid evolution and addition/deletion of zinc fingers (that provide the basis for hybrid sterility among *M. musculus* strains [15]) are thus recurrent across rodent evolution.

We also inferred evolutionary rates for each codon from an alignment of every *Prdm9* zinc finger from all of these 13 rodent species. Rates for three sites (sites −1, 3 and 6), together with a fourth (site −2), greatly surpass the neutral rate with values of *d_N_/d_S_* up to 8 (Figure 3C). These ratios greatly exceed those found for corresponding positions in other mammalian zinc finger genes [28]–[30]. These three positions (namely −1, 3 and 6) correspond exactly to the positions known to be involved in sequence-specific DNA-binding [31],[32]; structural studies have shown that amino acids within the zinc finger α-helix at positions −1, 3 and 6 make contacts with bases 3, 2 and 1 in the primary DNA strand respectively, whilst the amino acid at position 2 interacts with the complement of base 4 [33]. Thus the finding that positive selection on residues −1, 3 and 6 indicates that it has specifically acted to alter DNA-binding preferences encoded by *Prdm9*.

### Rapid evolution of *PRDM9* in primates

Based on our findings in rodents, we next undertook a survey of *PRDM9* divergence in the primate lineage to ask whether the extraordinary evolution of *Prdm9* was limited to rodents alone. In humans, there appear to be two genes that are orthologous to a single mouse *Prdm9*, suggesting a recent gene duplication [34],[35]. These two genes, *PRDM7* and *PRDM9*, are found at chromosomal locations 16q24.3 and 5p14, respectively. It is clear that since the gene duplication *PRDM7* has acquired distinct tissue-specific patterns of expression and has undergone major structural rearrangements, dramatically altering the number of encoded zinc fingers (2 in macaques, 5 in orangutans) while diverging from ancestral patterns of transcript splicing [34]. Furthermore, there is evidence for a frame-disruption affecting *PRDM7* in some humans. Consequently, we do not investigate *PRDM7* further in this report.

**Figure 4:**
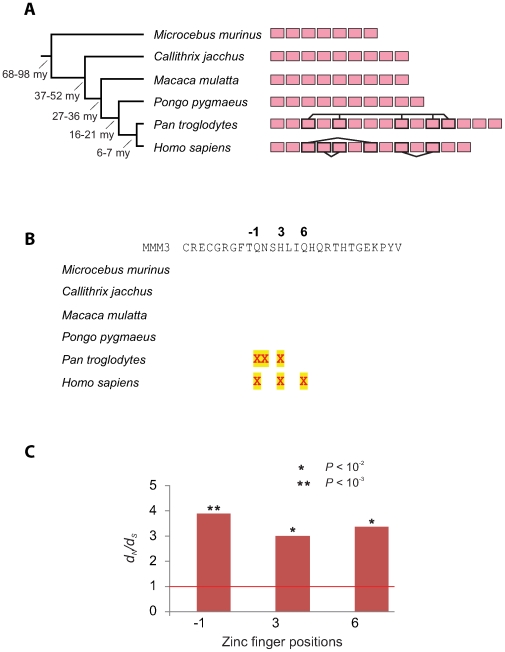
Concerted evolution and positive selection among primate *PRDM9* genes. (A) *PRDM9* C-terminal zinc fingers for 6 primate species are shown as pink rectangles. Zinc fingers whose nucleotide sequences are identical are joined by solid lines. Zinc fingers with identical sequences from the same species are consistent with gene conversion and/or intra-exon duplication. (B) Predicted positively selected sites in *Prdm9* from divergent primate lineages. Positive selection was inferred for each species [27] from intra-species *Prdm9* zinc finger sequence alignments. Positively selected sites (*P*<0.05 after multiple testing correction) are shown mapped to the third mouse Prdm9 zinc finger sequence (MMM3) as shown in Figure 3A. (C) Estimated *d_N_/d_S_* values at three zinc finger positions (namely −1, 3, and 6) in a comparison of zinc fingers from all primates for which there exists strong evidence of positive selection [27]. The *P*-values shown have been corrected for multiple testing. Common names to species are listed in the legend to Figure 6.

Primate *PRDM9* appears to show a large variation in numbers of zinc fingers in its C-terminal array similar to what we found in rodents (Figure 2A). Chimpanzee, orangutan, rhesus macaque and marmoset *PRDM9* genes encode 15, 10, 9, and 9 C-terminal zinc fingers as opposed to 13 in human *PRDM9* (Figure 4A). As in rodents, primate zinc fingers also show evidence for concerted evolution. For example, there are three identical pairs out of the C-terminal array of 13 zinc fingers encoded by human *PRDM9*.

When we compared the *PRDM9* gene sequence between humans and chimpanzees, we found the nucleotide divergence to be 7.1%, over 5-fold higher than the divergence observed genome-wide (1.23% [36]) although the high degree of concerted evolution complicates this human-chimpanzee ortholog comparison. However, it does appear that much of the divergence has resulted from a combination of positive selection and concerted evolution. Estimated *d_N_/d_S_* values for positions −1, 3 and 6 of human *PRDM9* zinc fingers are 12.6, 9.9 and 13.9 respectively, substantially greater than 1. Indeed, either by a species-specific zinc finger analysis (Figure 4B) or by a pooled analysis of all primate *PRDM9* encoded zinc fingers (Figure 4C), we find strong evidence for positive selection at these positions.

**Figure 5:**
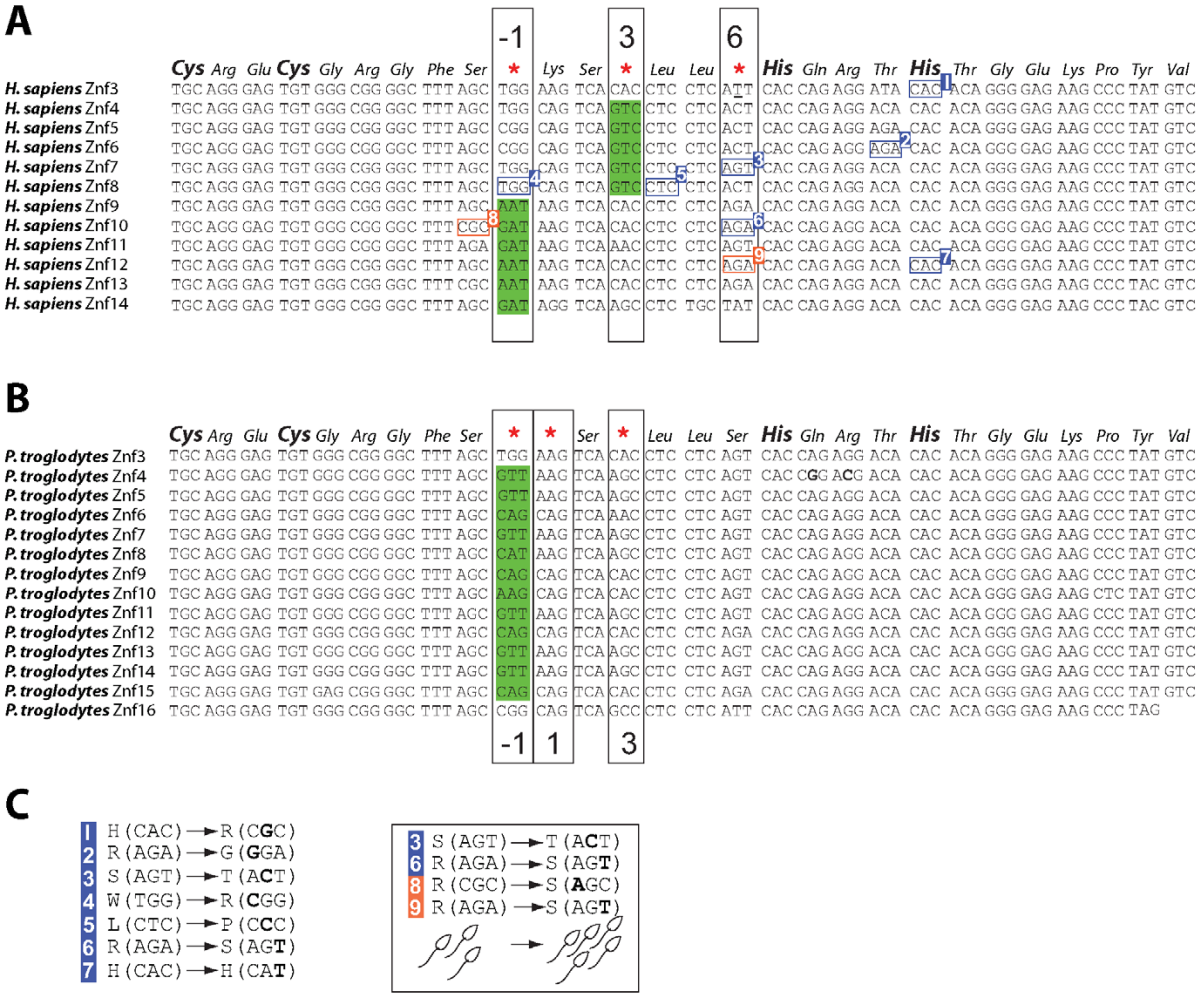
Sequence divergence and diversity among human and chimpanzee *PRDM9* zinc finger sequences. (A) Multiple sequence alignment of human (*Homo sapiens*) *PRDM9* zinc finger sequences, with positively selected positions (*P*<0.05, after multiple testing correction) indicated by red asterisks interspersed among a consensus amino acid sequence. Positions −1, 3, and 6 (numbered relative to the start of the zinc finger α–helix) that represent sequence-variable positions frequently involved in DNA binding are also indicated. Codons highlighted in green are not found at the same position in any chimpanzee *PRDM9* zinc finger. (B) Multiple sequence alignment of chimpanzee (*Pan troglodytes*) *PRDM9* zinc finger sequences with a predicted positively selected site indicated as in panel (A). Note that several chimpanzee *PRDM9* zinc finger codons (highlighted in green) at positions −1 and 3 are unique to this species, relative to humans (A). (C) Numbered and boxed codons in panel (A) contain human nonsynonymous SNPs. SNPs numbered 1–7 were identified in this study among 50 Chinese individuals whilst heterozygous SNPs numbers 3, 6, 8, and 9 are significantly enriched among fertile, as opposed to infertile, males in the study by Irie et al. [18].

Our findings suggest that positive selection and concerted evolution have directly and dramatically altered DNA-binding specificity of the encoded PRDM9 protein in primates as was observed in rodents. For instance, for 12 of the 15 C-terminal array of chimpanzee *PRDM9* zinc fingers, codons at position −1 are not found in any human *PRDM9* zinc finger at the same position; similarly, 6 human zinc fingers have codons at this position that are not present in the chimpanzee ortholog (Figure 5A and 5B). Like in rodents (Figure 2 and Figure 3), the *PRDM9* genes of closely related primate species are differentiated not only by the numbers of zinc fingers they encode, but also by species-specific codons, particularly at key positions that dictate DNA-binding specificity (Figure 4 and Figure 5).

### *PRDM9* evolution in humans

We next investigated whether positive selection on *PRDM9* had left population genetic signatures of selection that still remained evident among modern humans. Each of the two methods we employed exploits SNP data and accounts for issues concerning population structure and growth (see Materials and Methods). Particularly recent selective sweeps are characterized by long extents of linkage disequilibrium (LD) that ensue when the haplotype carrying the advantageous allele rises in frequency more rapidly than a neutral allele. Conversely, tests based on this characteristic are particularly sensitive for detecting recent episodes of positive selection [37]. Looking at patterns of LD, we did not find evidence for very recent selective sweeps at *PRDM9*. In our test we computed the maximum correlation coefficient (*r^2^*) between SNP pairs spanning the *PRDM9* locus, and compared these to the empirical distribution of this statistic across the genome. These maximum *r^2^*-statistics were not significantly different from the background (*p* values of 0.24, 0.23 and 0.24 for the African, European and Japanese/Chinese population panels).

Since tests based on long extents of LD or haplotypes are sensitive for very recent sweeps [37] only, while tests based on Tajima's *D* maintain power until some time after fixation of the advantageous allele [38], we also used a Tajima's *D* estimate to investigate whether polymorphisms linked to *PRDM9* exhibit an unusual population frequency spectrum. When an advantageous allele has risen to fixation, the extended haplotype associated with it will, for a considerable time thereafter, carry young and low-frequency polymorphisms, which may be observed as a reduction of Tajima's *D*, defined as the scaled difference of two estimators of heterozygosity which are identical under the standard neutral model [39]. There are significant caveats to the calculation of Tajima's *D* from genotyping data which bias against the recovery of low frequency SNPs. The Perlegen genotyping data have been shown to provide useful Tajima's *D* statistics after empirically accounting for this ascertainment bias [40],[41]. Using these methods, we calculated Tajima's *D* at the *PRDM9* locus [41] in African Americans (*D* = −0.130; *p* = 0.038), European Americans (*D* = −0.259, *p* = 0.068), and Asian Americans (*D* = 1.7). With the caveat that there might be uneven distribution of ascertainment biases across the genome, there appears to be weak evidence for a recent selective sweep in African Americans. In contrast to *PRDM9*, Tajima's *D* provides no evidence for recent sweeps in any of the three populations at the *PRDM7* locus.

We were interested in using intraspecies human polymorphisms to gain further insight into the evolutionary forces that drive the concerted evolution of *PRDM9*. To this end, we sequenced the terminal *PRDM9* zinc finger sequences from 50 Han Chinese individuals, seeking sequence polymorphisms that might have arisen by gene conversion. Under gene conversion, we would expect to observe a nucleotide polymorphism in one zinc finger that is identical to its fixed paralogous base in another. We observed 7 codons containing single nucleotide polymorphisms (SNPs; blue rectangles in Figure 5A and 5C). Of these, 4 (numbered 1, 2, 5 and 7 in Figure 5A and 5C) represent changes to codons that are not represented among any of the remaining zinc finger sequences and thus are unlikely to have arisen by gene conversion. The remaining 3 changes are to codons that are also present in at least one paralogous position within the other zinc fingers. A separate study identified 17 non-synonymous SNPs within human *PRDM9* zinc fingers, of which 13 showed evidence for having arisen by gene conversion from paralogous sequences [18]. We infer, therefore, that non-allelic gene conversion has contributed to the rapid evolution of primate *PRDM9*, and this provides a likely mutational mechanism for many other *PRDM9* orthologues.

What are the functional consequences of these non-synonymous SNPs in *PRDM9*? Two recent genetic association studies have investigated PRDM9 SNPs and their association with azoospermia. The first study [19] did not find correlated SNPs in the C-terminal zinc fingers. However, a second study found that individual nonsynonymous SNPs in the zinc finger domain are associated with a significantly decreased risk of infertility [18]. For instance, human non-synonymous SNPs (labelled 3, 6, 8 and 9 in Figure 5A and 5C) are associated with decreased risk of sterility in a cohort of Japanese men [18], of which two (numbers 3 and 6) were found among the 50 Han Chinese individuals we sequenced. In addition, 3 out of 4 non-synonymous SNPs associated with fertility are found at zinc finger position 6, a site predicted to determine DNA-binding specificity and which we show has evolved under positive selection in human *PRDM9* (Figure 4 and Figure 5A). Surprisingly, in each instance, the ‘minor’ allele at each position is associated with protection against sterility in Japanese men [18]. Intriguingly, in both studies, the effect on ameliorating azoospermia or oligospermia was manifest even in the heterozygous condition [18],[19], suggesting that *PRDM9*'s effect is semi-dominant (consistent with results of hybrid sterility seen in mouse *Prdm9*). In a situation where a minor allele provides a protective benefit against sterility, we might expect that high frequency retention of these alleles would be favored by balancing selection in this population. Consistent with this expectation, we point out that Asian American individuals had a striking Tajima's *D* of +1.7 in contrast to the negative Tajima's *D* in the other two populations in the Perlegen dataset, although this statistic by itself is not strong evidence of balancing selection given the ascertainment bias.

### Rapid evolution of *Prdm9* is an ancient feature in metazoans

**Figure 6:**
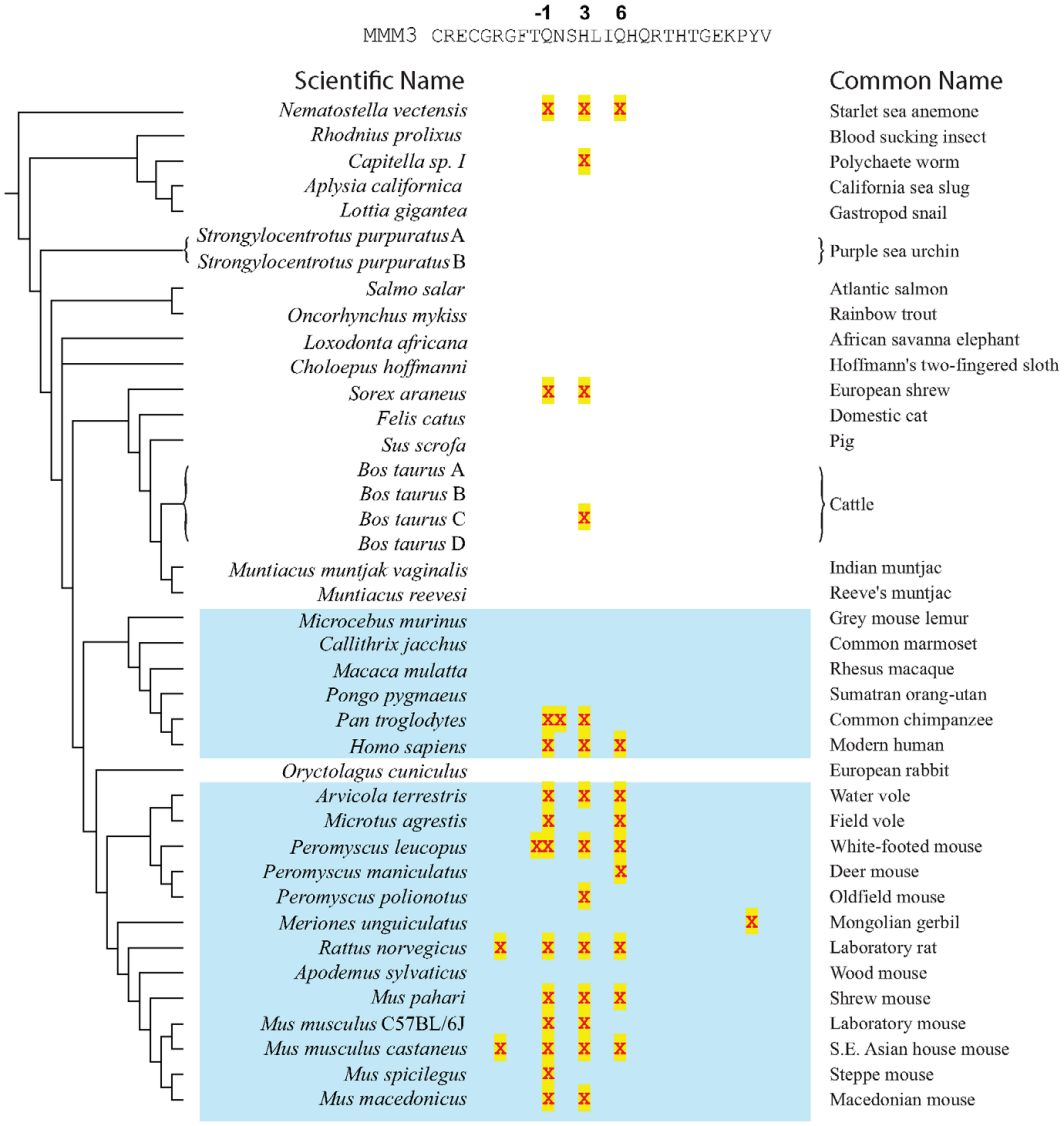
Predicted positively selected sites in *Prdm9* from divergent metazoan lineages. Results for previously presented rodent and primate lineages are also shown here for comparison (blue shading). Positive selection was inferred for each species [27] from intra-species *Prdm9* zinc finger sequence alignments. Positively selected sites (*P*<0.05 after multiple testing correction) are shown mapped to the third mouse Prdm9 zinc finger sequence (MMM3). The majority of positively selected sites, across 700 million years of divergence from sea anemone to mammals, fall at positions −1, 3, and 6. The inferences of positive selection for *Capitella* were made on the basis of three sequences on separate unassembled genomic scaffolds. Despite their high sequence similarity, multiple uncorrelated point substitutions, especially among the zinc fingers, suggest that they may represent allelic copies or rapidly diverging paralogues.

The two evolutionary themes (concerted evolution and positive selection) that typify *PRDM9* evolution in primates and in rodents also have occurred recurrently across metazoan evolution (summarized in Figure 6). For instance, we found evidence of concerted evolution among *Prdm9*-encoded zinc fingers in the sea anemone *Nematostella vectensis*, the gastropod snail *Lottia gigantea*, and the polychaete worm *Capitella* sp. I (Figure S3, S4, S5), organisms that last shared a common ancestor with mammals approximately 700 million years ago [42]. In addition, we find strong evidence of positive selection in zinc fingers of *N. vectensis Prdm9* for the same 3 positions (namely, −1, 3 and 6) also identified from analyses of rodent and primate lineages (summarized in Figure 6). Estimated *d_N_/d_S_* values for these positions were exceptionally high, ranging between 25 and 32. A single codon of the *Capitella* worm *Prdm9* zinc fingers also shows evidence of positive selection (Figure 6). Thus, even early branching metazoans show strong evidence of both concerted evolution and positive selection within *Prdm9*-encoded zinc fingers.

Concerted evolution is also apparent in *Prdm9* zinc fingers for many mammals including elephants (*Loxodanta africana*), cats (*Felis catus*), common shrews (*Sorex araneus*), cattle (*Bos taurus*), muntjak deer (*Muntiacus reevesi* and *Muntiacus muntjak vaginalis*), bats (*Myotis lucifugus*) and rabbits (*Oryctolagus cuniculus*) (data not shown). It is also evident among the zinc fingers of *Prdm9* from the Atlantic salmon (*Salmo salar*) and the rainbow trout (*Oncorhynchus mykiss*). Of the four complete zinc fingers in rainbow trout *Prdm9*, two are identical in nucleotide sequence, and the remaining pair are more closely-related to each other than they are to those of *Prdm9* for the Atlantic salmon (Figure S6), with which it last shared a common ancestor approximately 20 million years ago [43]. Evidence for positive selection is, however, less compelling outside of these fish, the sea anemone, rodents and primates. This is perhaps owing to the stringent multiple testing correction we employed, especially in cases where there are insufficient zinc fingers to obtain significant power for this kind of analysis (see Materials and Methods).

Despite strong evidence of concerted evolution and/or positive selection in many metazoan *Prdm9* sequences, this pattern is not universal across all metazoans. In comparisons of *Prdm9* in other ray-finned fishes (including *Danio rerio*) and in tunicates (including *Ciona intestinalis*), we found no evidence for either concerted evolution or positive selection within their zinc fingers. Among mammals, we found two homologs of *Prdm9* in the platypus *Ornithorhynchus anatinus*, but evidence for neither concerted evolution nor positive selection. When we investigated the *Prdm9* ortholog in the marsupial *Monodelphis domestica* and the nematode *Caenorhabditis elegans*, we were surprised to find a complete loss of all zinc fingers. Despite *Prdm9* being essential for fertility in mice, *Prdm9* appears lacking in chicken (*Gallus gallus*), frog (*Xenopus tropicalis*) and fly (*Drosophila melanogaster*) genomes, while the dog (*Canis familiaris*) genome has acquired multiple disruptive mutations (“pseudogenization”) within its *Prdm9* ortholog [44]. This either implies that *Prdm9* function in meiosis is carried out by another gene in these lineages, or that *Prdm9's* essential function in meiosis is itself lineage- or species-specific.

## Discussion

### Rapid evolution of DNA–binding specificity and insights into *Prdm9* function

Our finding of recurrent and dramatic episodes of rapid evolution of *Prdm9* in different lineages indicates that the protein-DNA interface at which Prdm9 acts, has frequently altered between, and within, species. These evolutionary observations allow us to revisit some key models of *Prdm9* function and how its divergence might give rise to hybrid sterility. The currently prevailing model is that *Prdm9* encodes a transcription factor for euchromatic genes during meiosis. Mouse *Prdm9 (Meisetz)* was first discovered for its essential role in meiotic prophase of both male and female meiosis [17]. Its SET domain was later found to catalyse the specific transition from di- to tri-methylation of the Lysine-4 residue on histone H3 (H3-K4), an activity that is characteristically associated with transcriptional activation [45]. Indeed, by tethering experiments, *Prdm9* was shown to be able to activate transcription. Furthermore, in *Meisetz^−/−^* testes, the transcriptional regulation of close to 125 genes was disturbed. Thus, *Prdm9 (Meisetz)* was proposed be a master transcriptional regulator of entry into meiosis in mammals, and all data including the intriguing association with human azoospermia [18],[19] are consistent with this view [17].

However, the accelerated evolution of the Prdm9-DNA interface challenges whether Prdm9's only, or even primary, role is a transcription factor for euchromatic genes. Such a function would leave unexplained why *cis*-acting (promoter) sequences to which Prdm9 binds, would be subject to repeated positive selection over the long time course of metazoan evolution. Rapid evolution at the protein-DNA interface would be especially disfavoured if it was required for fertility. We cannot formally rule out the unprecedented possibility that a transcription factor may evolve rapidly in concert with all of its (at least 125 [17]) *cis*-acting binding sites if indeed Prdm9 directly mediates the transcription activation of meiotic promoters. However, in general, the larger the number of *cis*-acting sequences that Prdm9 has to bind, the more its DNA-binding would be expected to be evolutionarily constrained which, we suggest, argues against its primary role as a transcription factor.

### An alternative model for *Prdm9* function

We considered the possibility that the rapid evolution of *Prdm9* was actually required for, rather than an impediment to, its function. One of the strongest observations in favor of the transcription model was the fact that the SET domain catalyzed transition from di- to tri-methyl H3-K4, a chromatin mark most often associated with transcriptional activation. And yet, this chromatin mark is not unique to transcriptional activation. Indeed, the same transition from di- to tri-methyl H3-K4, distinguishes canonical H3-nucleosomes at centromeric *versus* pericentric heterochromatic regions at mitotic centromeres of organisms as diverse as flies and humans [46]. Inactivation of a centromere on a human artificial chromosome directly results in loss of H3-K4 dimethylation and accumulation of H3-trimethylation [47].

**Figure 7:**
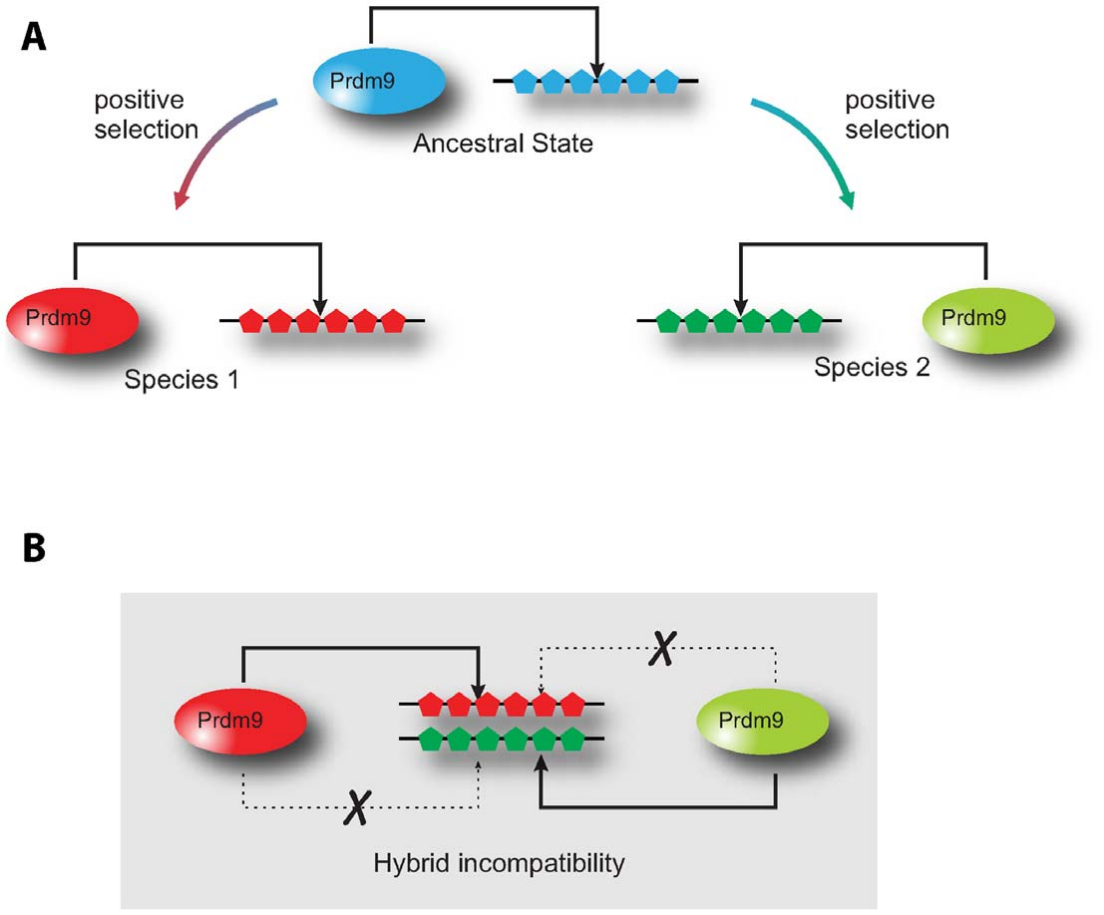
A novel satellite–DNA binding model for Prdm9 in hybrid sterility. (A) Prdm9 could serve as a satellite-DNA binding protein that facilitates its heterochromatinization. Hybrid sterility ensues when the sterility-associated Prdm9 protein (blue) cannot bind to “newly expanded” satellite DNA repeats (red or green) potentially at pericentric regions of chromosomes that arose due to centromere-drive [63]. (B) Under this model, in isolated populations, satellite–DNAs diverge quickly by “centromere-drive” and Prdm9 DNA–binding specificity evolves rapidly to suppress this drive. However, in hybrid males, inappropriate localization of Prdm9 to diverged DNA–binding satellites from other species would result in either inappropriate chromosome condensation (as shown) or compromised centromere function (not shown), either of which would result in male sterility.

We hypothesize that Prdm9's essential role in meiosis is directly related to its ability to bind rapidly-evolving DNA elements. While we do not know the identity of these DNA elements, we speculate that Prdm9 may function by binding directly to repetitive DNA sequences that are found at pericentric and centromeric regions (Figure 7A). Such repetitive DNA sequences (or ‘satellite repeats’) evolve exceedingly rapidly across multiple lineages [48]–[52]. It has been previously proposed that this rapid evolution results from centromere-drive [53],[54], a process in which meiotic products compete during female meiosis for retention in the egg *versus* exclusion as polar bodies. The genetic opportunity to ‘cheat’ during female meiosis is the evolutionary thread common among many repetitive DNA elements [55]–[58]. Further, DNA-binding proteins are thought to rapidly evolve their DNA-binding specificity to suppress this ‘meiotic drive’ [59]–[63]. Under this model, rapid changes in satellite-DNA sequences potentially ensuing from centromere-drive are followed by positive selection of non-synonymous substitutions within Prdm9 DNA-binding determinants to counter the deleterious effects of the meiotic (centromere) drive process. This would explain not only the rapid evolution and retention of *Prdm9* in most metazoans but also the loss of *Prdm9* genes in some lineages, when a second satellite-DNA binding protein may have taken over this suppressor function.

A recent study on the Drosophila *OdsH* hybrid sterility gene provides interesting parallels to the *Prdm9* study [64]. Due to its evolutionary descent from the *unc-4* transcription factor [11], *OdsH* was also believed to be a transcription factor. Since the DNA-binding homeobox domain had undergone rapid evolution, hybrid sterility was proposed to result from altered gene expression in *Drosophila* testis [65], much the same as it has been suggested for *Prdm9*. However, functional analyses of OdsH revealed it to function as a heterochromatin-binding protein, with altered DNA binding resulting in altered heterochromatic localization and chromosome decondensation [64]. A transcription factor function of *Prdm9* (like in *OdsH*) may be directly tied to a chromosome decondensation function. Indeed, work from a number of model systems especially the fission yeast *Schizosaccharomyces pombe* has revealed that transcription of heterochromatic repeats is a prequel and often a pre-requisite for the deposition of heterochromatin-specific histone modifications and proteins required for transcriptional silencing and condensation [66]–[69]. Prdm9 binding to satellite-DNA may facilitate its heterochromatinization by virtue of its transcriptional activity (Figure 7A), and alterations of Prdm9's binding specificity could allow it to act on a wider array of satellite-DNAs, consistent with its semi-dominant effect in hybrid sterility and human azoospermia. The chromosome decondensation and synapsis defects in male meiosis observed in sterile hybrids between *M. m. musculus* and *M. m. domesticus* species [15] would be explained by an inability to correctly bind and package satellite DNA (Figure 7B). Indirect consequences of such decondensation could be the transcriptional misregulation of some genes, as observed in *Prdm9^−/−^* mice [17]. Alternatively, ‘mismatched’ binding of Prdm9 to centromeric satellite-DNA repeats would result in their inappropriate heterochromatinization, again leading to chromosome condensation defects and male sterility. Under this model, mismatched Prdm9-satellite DNA configurations would be predicted to result in sterility only in hybrid males, but not in hybrid females [53]. We would like to emphasize the current absence of functional data to support such a hypothesis. However, the precedence provided by the *OdsH* study [64] and the consistent rapid evolution seen at Prdm9's DNA-binding interface provides a simple, testable explanation for the onset of highly context-specific hybrid sterility. Variation in *Hst1 (Prdm9)* occasions a genetic incompatibility between the Prdm9 DNA-binding protein encoded by this locus and the satellite DNAs to which Prdm9 binds (or fails to bind). The finding that human azoospermia is rescued by heterozygous *PRDM9* alleles [18],[19], including some that alter DNA-binding preferences, further suggests that a reduced repertoire of satellite-DNA binding ability may be responsible for the meiotic arrest at pachytene seen not only in the hybrid mice species [15], but also in the *Prdm9* ^−/−^ mice [17], a possibility that directly lends itself to genetic and cytological scrutiny.

The proposal that episodes of meiotic drive and suppression drive hybrid incompatibilities is not new [70],[71]. Indeed, cryptic meiotic-drive suppressor systems have been uncovered by introgression analyses between different *Drosophila* species [72]. Moreover, recent studies of hybrid inviability amongst *Drosophila* species have revealed the very likely role that pericentric heterochromatin plays in the manifestation of genetic incompatibility [73],[74]. While the molecular function of Prdm9 remains to be fully elucidated, our findings directly implicate the Dobzhansky-Muller incompatibility underlying *Prdm9*-mediated sterility as residing at a rapidly evolving protein-DNA interface.

### Recurrent rapid evolution of the *Prdm9* hybrid sterility gene

The onset of interspecies hybrid incompatibilities is widely believed to ensue as the by-product of acquired genetic differences in geographically isolated populations. This process can be imagined to take place in the absence of any selective pressure, purely by genetic drift [75]. However, the accumulation of genetic incompatibilities is more likely with accelerated evolutionary change, especially if recurrent genetic conflicts were driving the divergence. Consistent with this, many hybrid incompatibility genes for both sterility and inviability are associated with dramatic episodes of positive selection [11],[73],[76].

Here, we have shown that the *Prdm9* gene, which was identified as a hybrid sterility gene in mice [15], has evolved rapidly due to the dual forces of concerted evolution and positive selection. This rapid evolution is seen not just across the rodent lineage, but also in primates and especially humans, whereby some alleles at positively selected sites are associated with male sterility via azoospermia due to meiotic arrest. Strikingly, rapid evolution of *Prdm9* is observed in some fish, in the sea anemone and a polychaete worm and thus, parsimoniously, is an ancestral feature of metazoan evolution, an evolutionary period spanning 700 million years. This recurrent evolution of *Prdm9* is in stark contrast to both the *Ovd* and *OdsH* hybrid sterility gene in *Drosophila*, which appear to have evolved rapidly only in isolated lineages in which its role in hybrid sterility is manifest [11],[13] whereas the gene transposition of *JYalpha* is also highly lineage-specific [12]. From sequenced transcripts, *Prdm9* is known to be expressed in male and female germ-line tissues across diverse metazoans such as trout, cattle, pig, sea urchin, and gastropod snail (accessions: CR372724, EF432552, EW634943, AM222434 and CAXX2975) in line with its previously described expression profile for mouse [17].

Hybrid sterility has been shown to arise from the simple deletion or insertion of a zinc finger domain in *Prdm9* in mice [15]. The loss or gain of a single zinc finger is among the least perturbing of all changes in zinc finger number and sequence we have observed. For example, even closely related species, such as humans and chimpanzees, or bank and field voles, or rainbow trout and Atlantic salmon, differ much more dramatically at DNA-binding positions of their Prdm9 zinc fingers (Figure 3, Figure 4, Figure 5, Figure S2, Figure S6). Moreover, findings from human genetic association studies demonstrate that even individual amino acid changes in PRDM9 can affect male fertility even within species [18]. Finally, recent studies clearly demonstrate that *Hst1 (Prdm9)* associated genetic incompatibilities have evolved independently and are polymorphic in both *M. m. musculus* and *M. m. domesticus* mouse subspecies [16]. Our study has found even more radical alterations within *Prdm9* zinc fingers than are observed in the *M. m. musculus* x *M. m. domesticus* cross. These changes, by themselves, may not be sufficient to result in reproductive isolation, as incompatibilities with a (as yet unknown) rapidly evolving DNA component would be required for hybrid sterility. In addition, hybrid sterility is clearly affected by multiple other loci [6],[7] whose discovery will lend further insight into the biological forces behind hybrid sterility. Nevertheless, our findings of recurrent rapid evolution of *Prdm9* suggest its candidacy as a postzygotic hybrid sterility gene in other metazoan taxa.

## Materials and Methods

### Predicting *Prdm9* genes

*Prdm9* genes, and their 3′ (carboxy-terminal) arrays of zinc fingers (Figure 1, Figure 2, Figure 3, Figure 4, Figure 5, Figure 6), were predicted from genome sequences available from UCSC, Ensembl and JGI genome browsers. Additional *Prdm9* sequences were identified from the interrogation of nucleotide sequence databases using TBLASTn. Prediction of 3′ *Prdm9* zinc finger sequences is greatly facilitated by their presence in the single 3′ terminal coding exon in all species. Orthology of *Prdm9* sequences was confirmed using phylogenetic analysis [44], by consideration of the KRAB-SET-zinc finger domain architecture that is conserved among many but not all (including some fish, *C. elegans* and *Monodelphis*) Prdm9 proteins (see text), and by reciprocal best BLAST hits. Details of *Prdm9* gene predictions from all species investigated are provided in Dataset S1.

### Sequencing of *Prdm9* zinc fingers in multiple rodent species

In addition to genomic data obtained for *Mus musculus* and *Rattus norvegicus*, sequencing of the final exon of *Prdm9* was performed from genomic DNA purified from reproductive tract tissue from a total of 11 additional (sub-)species: *Mus musculus castaneus*, *Mus macedonicus*, *Mus spicilegus*, *Coelomys pahari*, *Apodemus sylvaticus*, *Meriones unguiculateus*, *Peromyscus leucopus*, *Peromyscus maniculatus*, *Peromyscus polionotus*, *Microtus agrestis* and *Arvicola terrestris*. PCR products were amplified using primers designed from the most highly conserved regions from mouse and rat genomic sequence flanking the last exon; either: *Mus*-*Prdm9*-F1 5′ CAAAGAACAAATGAGATCTGAG or *Mus*-*Prdm9*-F2 5′ AGAACAGGCCAGACAACAAAT with *Mus*-*Prdm9*-R1 5′ GTCTT(C/T)CTGTAATTGTTGAGATG or *Mus*-*Prdm9*-R2 5′ GCT(G/A)TTGGCTTTCTCATTC. Products were amplified using the proof-reading *Pfx* DNA polymerase (Invitrogen), purified from agarose gels using the Qiaquick gel purification kit (Qiagen) and sequenced in both directions from 2 or more independent amplification reactions. Sequence traces were initially curated and assembled using Chromas 2.0 (http://www.technelysium.com.au/chromas.html) and Bioedit (http://www.mbio.ncsu.edu/BioEdit/bioedit.html). Genbank accessions are provided in Dataset S2.

### Sequencing of *PRDM9* zinc fingers from human and chimpanzee samples

Sequencing of the zinc finger repeat domain of *PRDM9* was performed from the genomic DNA of 50 Chinese normal control samples. PCR amplification, purification and sequencing was carried out as above using the primers Hs-*PRDM9*-F 5′-GGCCAGAAAGTGAATCCAGG-3′ and Hs-*PRDM9*-R 5′-TGAAGCCACCTCACACAGCTG-3′. Products were gel purified and A-tailed prior to sub-cloning into the pCR4-TOPO vector (Invitrogen). T7 and T3 vector primers were used to sequence mini-prep DNA from positive clones. Genbank accessions are provided in Dataset S2.

Chimpanzee (*Pan troglodytes*) PRDM9 C-terminal zinc fingers were sequenced by PCR using the primers Pt-*PRDM9*-F 5′-GCCTGACCAAAACATCTACCCTGACC-3′ and Pt-*PRDM9*-R 5′-GTCATGAAAGTGGCGGATTTG-3′. PCR products were both directly sequenced as well as cloned into the pCR4-TOPO vector (Invitrogen) and six independent clones sequenced using vector-specific primers. The genomic DNA sample was obtained from Coriell (ID#NG03448). The Genbank accession can be found in Dataset S2.

### Prediction of positively selected codons

For the prediction of positively selected sites, we included all zinc finger sequences from the 3′ terminal array only if they were complete (28-codon) and retained, at conserved positions, two cysteine and two histidine residues expected to coordinate a single Zn^2+^ ion. This excludes, for example, the first two zinc finger motifs in primates and rodents. Phylogenetic trees for each multiple alignment were constructed by applying the Fitch-Margoliash criterion to distance matrices of synonymous substitutions per synonymous site (*d_S_*) as calculated by the codeml programme [77],[78]. Tree topologies were accepted if they were corroborated by phyml [79] and treebest (http://treesoft.sourceforge.net/treebest.shtml) programs.

Amino acid sites under positive selection were inferred using “site likelihood method” (SLR) [27] with *p*-value thresholds of 0.05 after multiple testing correction. We observed that inferences of positive selection among sequence similar zinc fingers from the same species were sensitive to tree topology. Nevertheless, the use of alternative less-well supported topologies tended only to increase evidence for positive selection. As a result, we have, conservatively, used inferences from the most strongly supported tree.

SLR, and other maximum-likelihood approaches that take account of codon evolution, have proved reliable provided that assumptions in evolutionary models are not greatly violated. One such assumption is vertical inheritance without gene conversion, which is demonstrably violated for *Prdm9*. However, gene conversion is more likely to affect analyses of sequence-similar zinc fingers from the same species and is less of a factor in analyzing zinc fingers from all the rodent or primate clades due to the greater sequence divergences involved (for instance, all identical zinc fingers are essentially treated as one representative sequence in analyses). Our inferences of positive selection among all zinc fingers in rodent or primate clades (Figure 3C and Figure 4C) are accordingly the most robust to phylogeny variations and show high *d_N_/d_S_* values, and low and significant *p*-values.

### Tests for positive selection in the human population

Rapid fixation of an advantageous allele changes the pattern of polymorphisms around the locus under selection, and various methods have been developed to formally test whether such patterns are compatible with evolution under a neutral model. Other effects, such as geographical structure, population admixture, non-random mating, and varying population sizes, can also give rise to a departure from the neutral model, thereby confounding this analysis. To address this problem, here we use data from recent large-scale surveys of population variation that allow us to compare our observations to empirical, genomic distributions rather than to model-based predictions. This approach accounts for non-local genomic effects such as population structure and growth, at the expense of some loss of power.

Tajima's *D* values were acquired from the UCSC genome browser for American individuals of African, European and Asian ancestry populations [40]. These were computed at 10 kb intervals, each using 100 kb of data. Since both *PRDM7* and *PRDM9* span about 20 kb, we took the average of two neighbouring values. For the background distribution, averages were similarly computed for all neighbours.

To assess the existence of long haplotype blocks, we used HapMap data (public release 26). We computed derived allele frequencies (DAF) by polarizing using the chimpanzee genome. To avoid miscalls, we removed all potential CpG SNPs. Finally, we used *r*-squared values computed for SNPs at a minimum distance of 50 kb, as including more proximal SNPs which are often in strong LD would further reduce power. For any locus, we identified all pairs of SNPs spanning the locus that satisfied these filters; the maximum *r*-squared value among these pairs was taken as the observable for that locus. We computed this value for all genomic loci to create the empirical distribution. The entire procedure was done separately for each of the HapMap populations.

### Calculations of zinc finger sequence identities in the mouse genome

Clusters of zinc finger repeats (Figure 2B) were identified in each of six possible reading frames of the mouse genome using the hmmsearch programme [80] and a hidden Markov model derived from the SMART domain resource [81]. We discarded all zinc finger clusters which show frameshift or stop codon disruptions, giving 473 putative open reading frames (ORFs). Within each ORF, zinc fingers which do not possess the canonical zinc finger Cys_2_His_2_ structure were excluded from subsequent comparisons. A multiple alignment of conceptual cDNA zinc finger sequences was constructed from peptide alignments using the MUSCLE programme [82]. Pairwise cDNA sequence alignments were calculated and the proportions of pairs which were higher than a given threshold calculated. Mouse *Prdm9* was an extreme outlier for zinc finger pairwise sequence identities greater than 90%, and also for other thresholds (data not shown).

## Supporting Information

Figure S1 Phylogenetic tree of rodent *Prdm9* zinc finger nucleotide sequences as inferred by phyml [76] version 3.0 (http://www.atgc-montpellier.fr/phyml/) and drawn using FigTree (http://tree.bio.ed.ac.uk/software/figtree). Zinc fingers are numbered sequentially from the C-terminal array. The exceptions are zinc fingers from the mouse and rat, whose numbers start from the first *Prdm9* zinc finger in exon 11. For species names see legend to Figure 6. Branches with Approximate Likelihood-Ratio Test values of over 0.75 are indicated with bold lines.
(6.18 MB EPS)

Figure S2 Multiple sequence alignment (shaded according to a 90% consensus) of *Prdm9* zinc finger sequences from the water vole (*Arvicola terrestris*) and field vole (*Microtus agrestis*).
(2.75 MB EPS)

Figure S3 Multiple sequence alignment (shaded according to a 90% consensus) of *Prdm9* zinc finger sequences from the sea anemone, *Nematostella vectensis*. These form part of a predicted gene (NEMVEDRAFT_v1g113856) that has been predicted from scaffold_120 of the *N. vectensis* v.1.0 genome assembly (Joint Genome Institute).
(2.64 MB EPS)

Figure S4 Multiple sequence alignment (shaded according to a 90% consensus) of 23 *Lottia gigantea Prdm9* zinc finger sequences. These have been predicted from scaffold 11 (bases 1507994-1510370) of the *Lottia* genome assembly (v1.0) produced by the Joint Genome Institute. This gene prediction is supported by an expressed sequence tag from *L. gigantea* male gonad (accession code FC692069).
(2.84 MB EPS)

Figure S5 Multiple sequence alignment (shaded according to a 90% consensus) of 11 *Capitella sp.I Prdm9* zinc finger sequences. These have been predicted from scaffold_236 of the *Capitella sp.I* v.1.0 genome assembly (Joint Genome Institute).
(2.95 MB EPS)

Figure S6 Multiple sequence alignment (shaded according to a 90% consensus) of *Prdm9* zinc finger sequences from the Atlantic salmon (*Salmo salar*; accession ACN10800, supported by ESTs CX352799, GE785155, EG785159, and EG785158) and from the Pacific Ocean rainbow trout (*Oncorhynchus mykiss*; contig generated from ESTs CR372724, CX253406 1305997, CX253405, CX252076, CX251898, CX251897, and CX252077).
(2.67 MB EPS)

Dataset S1 Multiple sequence alignment of *Prdm9* zinc fingers from all analysed species in FASTA format.
(0.09 MB PDF)

Dataset S2 Genbank accessions for rodent and human sequences
(0.04 MB DOC)
