# Supplementary material for: Accelerated Evolution of the Prdm9 Speciation Gene across Diverse Metazoan Taxa
Source: PLoS Genet. 2009 Dec 4;5(12):e1000753. doi: 10.1371/journal.pgen.1000753 (PMC2779102; doi:10.1371/journal.pgen.1000753)
Supplement: Dataset S1 — Multiple sequence alignment of Prdm9 zinc fingers from all analysed species in FASTA format. (0.09 MB PDF) [file pgen.1000753.s007.pdf]

>aplysia_californica__00
TGTGAGGTTTGTGGTGCTGGGTTTACACAGAGTAGAGACCTACAGAAGCACAAAAGAACACACACGGGAGAAA
AGCCTTACAGT
>aplysia_californica__01
TGTGAAGTTTGTGGTGCAGGGTTTACACAGTATGGCAACCTACAGAGTCACAAAAGAACACACACGGGAGAAA
AGCCTTACAAG
>aplysia_californica__02
TGTGAAGTTTGTGGTGCAAGGTTTGCACAGTATGGCAACCTACAGAGTCACAAAAGAACACACACGGGAGAAA
AGCCTTACAAG
>aplysia_californica__03
TGTGAAGTTTGTGGTGCAGGGTTTGCACGGAGTGGCGACCTACAGAGTCACAAAAGAACACACACGGGAGAAA
AGCCTTATAAG
>aplysia_californica__04
TGTGAAGTTTGTGGTGCAGGGTTTGCACACAGTGGCCACCTACAGAGTCACAAAAGAACACACACGGGAGAAA
AGCCTTACCAA
>aplysia_californica__05
TGTGAAGTTTGTGGTGCAGGGTTTACACAGTATGGCAACCTACAGAGTCACAAAAGAACACACACGGGAGAAA
AGCCTTATAAG
>aplysia_californica__06
TGTGAAGTTTGTGGTGCAGGGTTTGCACACAGTGGCCACCTACAGAGTCACAAAAGAACACACACGGGAGAAA
GGCCTTACAAG
>aplysia_californica__07
TGTGAAGTTTGTGGTGCAGGGTTTGCACACAGTGGCCACCTACAGCGTCACAAAAGAACACACACGGGAGAAA
AGCCTTACAAG
>aplysia_californica__08
TGTGAAGTTTGTGGTGCAGGTTTTGCACGGAGTGGCCACCTACAGAGTCACAAAAGAACACACACGGGAGAAA
AGCCTTACAAG
>aplysia_californica__09
TGTGAAGTTTGTGGTGCAGGGTTTACACAGTATGGCAACCTACAGAGTCACAAAAGAACACACACGGGAGAAA
AGCCTTACAAG
>aplysia_californica__10
TGTGAAGTTTGTGGTGCAGGGTTTACACAGTATGGCAACCTACAGAGTCACAAAAGAACACACACGGGAGAAA
AGCCTTACAAG
>apodemus_sylvaticus__01
TGCAGGGAGTGTGGGCGGGGCTTTACACAGAAGTCACACCTCAACCGTCACCAGAGGACACACACTGGGGAGA
AGCCCCATGTT
>apodemus_sylvaticus__02
TGCAGGGAGTGTGGGCGGGGCTTTACACAGAAGTCACACCTCAACCGTCACCAGAGGACACACACTGGGGAGA
AGCCCCATGTT
>apodemus_sylvaticus__03
TGCAGGGAGTGTGGGCGGGGCTTTACACTGAAGTCAAACCTCAACCGTCACCAGAGGACACACACAGGGGAGA
AGCCCTGTGTT
>apodemus_sylvaticus__04
TGCAGGGAATGTGGGCGGGCCTTTACACAGAAGTCAGACCTCATCCAGCACCAGAGGACACACACTGGGGAGA
AGCCCTATGTT
>apodemus_sylvaticus__05
TGCAGGGAGTGTGGGCGGGGCTTTACACAGAAGTCAAACCTCAACCAGCACCAGAGGACACACACTGGGGAGA
AGCCCTATGTT
>apodemus_sylvaticus__06
TGCAGGGAGTGTGGGCGGGGCTTTACACGGAAGTCACTCCTCATCCAGCACCAGAGGACACACACTGGGGAGA
AGCCCTATGTT
>apodemus_sylvaticus__07
TGCAGGGAGTGTGGGCGGGGCTTTACACAGAAGTCAGACCTCAACCGTCACCAGAGGACACACACAGGGGAGA
AGCCCTATGTT

>apodemus_sylvaticus__08
TGCAGGGAGTGTGGGCGGGGCCTTACACAGAAGTCAAACCTCATCCAGCACCAGAGGACACACACTGGGGAGA
AGCCCTATGTT
>apodemus_sylvaticus__09
TGCAGGGAATGTGGGCGGGGCTTTACACTGAAGTCAGACCTCATCCAGCACCAGAGGACACACACTGGGGAGA
AGCCCTATGTT
>apodemus_sylvaticus__10
TGCAGGGAGTGTGGGCGGGGCTTTACACGGAAGTCAGACCTCAACCGTCACCAGAGGACACACACAGGGGAGA
AGCCCTATGTT
>apodemus_sylvaticus__11
TGCAGGGAGTGTGGGCGGGGCTTTACACAGAAGTCAAACCTCATCCAGCACCAGAGGACACACACTGGGGAGA
AGCCCTATGTT
>apodemus_sylvaticus__12
TGCAGGGAATGTGGGCGGGGCTTTACACTGAAGTCAGACCTCATCCAGCACCAGAGGACACACACTGGGGAGA
AGCCCTATGTT
>arvicola_terrestris__02
TGCAGGGAGTGTGGGCGGGGCTTTACACGGAAGTCAGTCCTCATCCTTCACCAGAGGACACACACAGGGGAGA
AGCCCTATGTT
>arvicola_terrestris__03
TGCAGGGAGTGTGGGCGGGGCTTTACACAGAAGTCAGTCCTCATCAATCACCAGAGGACACACACAGGGGAGA
AGCCCTATGTT
>arvicola_terrestris__04
TGCAGGGAGTGTGGGCGGGGCTTTACACAGAAGTCACACCTCATCTTTCACCAGAGGACACACACAGGGGAGA
AGCCCTATGTT
>arvicola_terrestris__05
TGCAGGGAGTGTGGGCGGGGCTTTACACAGAAGTCACACCTCATCCTTCACCAGAGGACACACACAGGGGAGA
AGCCCTATGTT
>arvicola_terrestris__06
TGCAGGGAGTGTGGGCGGGGCTTTACATGGAAGTCAGTCCTCATCCTTCACCAGAGGACACACACAGGGGAGA
GGCCCTATGTT
>arvicola_terrestris__07
TGCAGGGAGTGTGGGCGGGGCTTTACACGGAAGTCACACCTCATCCTTCACCAGAGGACACACACAGGGGAGA
AGCCCTATGTT
>arvicola_terrestris__08
TGCAGGGAGTGTGGGCGGGGCTTTACACAGAAGTCACACCTCATCCTTCACCAGAGGACACACACAGGGGAGA
AGCCCTATGTT
>arvicola_terrestris__09
TGCAGGGAGTGTGGGCGGGGCTTTACACGGAAGTCAGTCCTCATCCTTCACCAGAGGACACACACAGGGGAGA
AGCCCTATGTT
>bos_taurus_BC149031_mRNA_01
TGTCATGTATGTAGGAAAGCCTTCAGTAAAAGTTCTAACCTTAGACGACATGAGATGATTCACACTGGAGTGA
AACCACATGGA
>bos_taurus_BC149031_mRNA_02
TGTCATCTTTGTGGAAAATCCTTCACTCATTGTTCTGACCTTAGAAAACATGAAAGAATTCACACTGGAGAGA
AATTATATGGA
>bos_taurus_BC149031_mRNA_03
TGTCATCTGTGTGGCAAAGCCTTCAGTAAAAGTTATAATCTTAGGCGACATGAGGTGATTCACACCAAAGAGA
AACCAAATGAA
>bos_taurus_BC149031_mRNA_04
TGTCATCTGTGTGGGAAAGCCTTTGCTCATTGTTCTGACCTTAGAAAACATGAGAGAACTCATTTTGGGGAGA
AACCATATGGA
>bos_taurus_BC149031_mRNA_05
TGCCACCTGTGTGGGAAGACTTTCAGTAAAACGTCTTACCTTAGACAACATGAGAGAACTCACAATGGAGAGA
AACCATATGGA

>bos_taurus_BC149031_mRNA_06
TGTCATCTATGTGGGAAGGCCTTCACTCATTGTTCTCACCTTAGAAAACATGAGAGAACTCACACTGGAGAGA
AGCCATATGAA
>bos_taurus_BC149031_mRNA_07
TGCCATCTATGTGGAAAAGCCTTCACTGAATCTTCTGTCCTTAGACGACATGAGAGGACTCACACTGGGGAGA
AGCCATATGAA
>bos_taurus_BC149031_mRNA_08
TGTCACCTGTGCTGGAAAGCCTTCACTGATTCTTCTGTCCTTAAGCGACACGAAAGAACTCACACTGGAGAGA
AACCATATGAA
>bos_taurus_BC149031_mRNA_09
TGTCACCTATGTGGGAAAACCTTCAATCACTCTTCTGTCCTTAGACGACATGAAAGAACTCACACAGGTGAGA
AACCATATGAA
>bos_taurus_BC149031_mRNA_10
TGCAATATATGTGGTAAAGCCTTCAATAGGAGTTATAACTTTAGATTGCATAAGAGAATTCACACTGGAGAGA
AACCATATAAA
>bos_taurus_EF432552_mRNA_01
TGCGGGGAGTGTGGGCAAAGCTTCAATCAGAAGTCCACTCTCATCACACACCAGAGGACACACACAGGGGAGA
AGCCCTATGTT
>bos_taurus_EF432552_mRNA_02
TGCGGGGAGTGTGGGCGAAGCTTCAATCAGAAGTCCACTCTCATCACACACCAGAGGACACACACAGGGGAGA
AGCCCTATGTT
>bos_taurus_EF432552_mRNA_03
TGCGGGGAGTGTGGGCGAAGCTTCAGTCAGAAGTCCACTCTCATCAAACACCAGAGGACACACACAGGGGAGA
AGCCCTATGTT
>bos_taurus_EF432552_mRNA_04
TGCGGGGAGTGTGGGCAAAGCTTCAATCAGAAGTCCACTCTCATCACACACCAGAGGACACACACAGGGGAGA
AGCCCTATGTT
>bos_taurus_EF432552_mRNA_05
TGCGGGGAGTGTGGGCAAAGCTTCAATCAGAAGTCCACTCTCATCACACACCAGAGGACACACACAGGGGAGA
AGCCCTATGTT
>bos_taurus_EF432552_mRNA_06
TGCGGGGAGTGTGGGCGAAGCTTCAGTCGGAAGTCCACTCTCATCACACACCAGAGGACACACAGA---
GAGAAGCCTTATGTT
>bos_taurus_chrX_85_77_01
TGTAAGGAGTGTGGGAAAAGCTTCAATGGGAGGTCAGATCTCACCAAACATAAGAGGACACACACAGGGGAGA
AGCCCTATGCT
>bos_taurus_chrX_85_77_02
TGTGGGGAGTGTGGGCGAAGCTTCAGTTTTAAGAAAAATCTCATCACACACAAGAGGACACACACACGGGAGA
AGCCCTATGTT
>bos_taurus_chrX_85_77_03
TGCAGGGAGTGTGGGCGAAGCTTCAATGAGAAGTCACGTCTCACCATACATAAGAGGACACACACAGGGGAGA
AGCCCTATGTT
>bos_taurus_chrX_85_77_04
TGTGGTGATTGTGGGCAAAGCTTCAGTTTGAAGTCAGTTCTCATCACACACCAGAGGACACACACAGGGGAGA
AGCCCTATGTT
>bos_taurus_chrX_85_77_05
TGTGGGGAGTGTGGGCGAAGCTTCAATGAGAAGTCACGTCTCACCATACATAAGAGGACACACACAGGGGAGA
AGCCCTATGTT
>bos_taurus_chrX_85_77_06
TGTGGTGATTGTGGGCAAAGCTTCAGTTTGAAGTCAGTTCTCATCACACACCAGAGGACACACACAGGGGAGA
AGCCCTATGTT
>bos_taurus_chrX_85_77_07
TGTGGGGAGTGTGGGCAAAGCTTCAATGAGAAGTCACGTCTCACCATACATAAGAGGACACACACAGGGGAGA
AGCCCTATGCT

>bos_taurus_chrX_85_77_08
TGTGGTGATTGTGGGCAAAGCTTCAGTTTGAAGTCAGTTCTCATCACACACCAGAGGACACACACAGGGGAGA
AGCCCTATGTT
>bos_taurus_chrX_85_97_01
TGCACGGAGTGTGGGAAAAGCTTCAATTGGAAGTCAGATCTCACCAAACATAAGAGGACACACTCAGAGGAGA
AGCCCTATGCT
>bos_taurus_chrX_85_97_03
TGTGGGGAGTGTGGGCAAAGCTTCAGTTTTAAGAAAAATCTCATCACACACCAGAGGACACACACAGGGGAGA
AGCCCTATGTT
>bos_taurus_chrX_85_97_04
TGCGGGGAGTGTGGGCGAAGCTTCAGTGAGAAGTCACGTCTCACCACACATAAGAGGACACACACAGGGGAGA
AGCCCTATGTT
>bos_taurus_chrX_85_97_05
TGTGGTGATTGTGGGCAAAGCTTCAGTTTGAAGTCAGTTCTCATCACACACCAGAGGACACACACAGGGGAGA
AGCCCTATGTT
>bos_taurus_chrX_85_97_06
TGCAGGGAGTGTGGGCGAAGCTTCAGTGTGATATCCAATCTCATCAGACACCAAAGGACACACACAGGGGAGA
AGCCCTATGTT
>bos_taurus_chrX_85_97_07
TGCAGGGAGTGTGAGCAAAGCTTCAGGGAGAAGTCCAATCTTGTCAGACACCAAAGGACACACACAGGGGAGA
AGCCCTATGTT
>callithrix_jacchus__01
TGCACGGAGTGTGGGCGGGGCTTTAGCCAGAAGTCAGTCCTCCTCAGTCACCAGAGGACACACACGGGGGAGA
AGCCCTATGTC
>callithrix_jacchus__02
TGCACGGAGTGTGGGCGGGGCTTTAGCCGGAAGTCAAACCTCCTCAGTCACCAGAGGACACACACGGGGGAGA
AGCCCTATGTC
>callithrix_jacchus__03
TGCAGGGAGTGTGGGCGGGGCTTTAGCCGGAAGTCAGCCCTCCTCAGTCACCAGAGGACACACACAGGGGAGA
AGCCCTATGTC
>callithrix_jacchus__04
TGCAGGAAGTGTGGGCGGGGCTTTAGCCAGAAGTCAAACCTCCTCAGTCACCAGGGGACACACACGGGGGAGA
AGCCCTATGTC
>callithrix_jacchus__05
TGCACGGAGTGTGGGCGGGGCTTTAGCCAGAAGTCACACCTCCTCAGTCACCAGAGGACACACACCGGGGAGA
AGCCCTATGTC
>callithrix_jacchus__06
TGCAGGAAGTGTGGGCGGGGCTTTAGCCAGAAGTCAAACCTCCTCAGTCACCAGAGGACACACACGGGGGAGA
AGCCCTATGTC
>callithrix_jacchus__07
TGCAGGGAGTGCGGGCGGGGCTTTAGCTTTAAGTCAGCCCTCCTCAGACACCAGAGGACACACACGGGGGAGA
AGCCCTATGTC
>callithrix_jacchus__08
TGCAGGGAGTGTGGGCGGGGCTTTAGCCGGAAGTCACACCTCCTCAGTCACCAGGGGACACACATAGGGGAGA
AGCCCTATGTC
>callithrix_jacchus__09
TGCAGGGAGTGTGGGCGGGGCTTTAGCCGGAAGTCAAACCTCCTCAGTCACCAGAGGATACACACAGGGGAGA
AGCCCTATGTC
>capitella_A_gene_01
TGCAGTGTATGCAATAAAGGATTCAGTCAATCTGGTCATCTGAAAAAACACACGAGGATTCACAGTGGTGAGA
AGCCATTCACA
>capitella_A_gene_02
TGCAGTGTATGCAAGAAAGGATTCAATCAATCTGGTGATCTGAAAAAACACATGAGGATTCACAGTGGTGAGA
AGCCATTCGCA

>capitella_A_gene_03
TGCAGTGTATGCAATAAAGGATTCAGTGATGCTGGCAATCTGAAAAAACACATGATGATTCACAGTGGTGAGA
AGCCATTCACA
>capitella_A_gene_04
TGCAGTGTATGCAATAAAGGATTCAGTGATGCTGGTCATCTGAAAACACACATGAGGATTCACAGTGGTGAGA
AGCCATTCGCA
>capitella_A_gene_05
TGCAGTGTATGCAATAAAGGATTCAGTGATGCTGGCAATCTGAAAAAACACATGATGATTCACAGTGGTGAGA
AGCCATTCACA
>capitella_A_gene_06
TGCAGTGTATGCAAGAAAGGATTCAATCGAGCTGATGTTCTGAAAACACACATGAGGATTCACAGTGGTGAGA
AGCCATTCACA
>capitella_A_gene_07
TGCAGTGTATGCAAGAAAGGATTCAATCAATCTGGTGATCTGAAAAAACACATGAGGATTCACAGTGGTGAGA
AGCCATTCACA
>capitella_A_gene_08
TGCAGTGTATGCAATAAAGGATTCAATCAATCTGGTGATCTGAAAAAACACATGAGGATTCACAGTGGTGAGA
AGCCATTCACA
>capitella_A_gene_09
TGCAGTGTATGCAATAAAGGATTCAATCAATCTGGTCATCTGAAAACACACATGAGGATTCACAGTGGTGAAA
AGCCATTCGCA
>capitella_A_gene_10
TGCAGTGTATGCAATAAAGGATTCAGTGATGCTGGCAATCTGAAAAAACACATGAGGATTCACAGTGGTGAGA
AGCCATTTACA
>capitella_B_gene_01
TGCAGTGTATGCAATAAAGGATTCAATCGAGCTGATGTTCTGAAAACACACATGAGGATTCACAGTGGTGAGA
AGCCATTCGCA
>capitella_B_gene_02
TGCAGTGTATGCAATAAAGGATTCAATCAATCTGGT---
CTGAAAACACACATGAGGATTCACAGTGGTGAGAAGCCATTCGCA
>capitella_B_gene_03
TGCAGTGTATGCAATAAAGGATTCAATCAATCTGGTTATCTGAAA---
CACATGAGGATTCACAGTGGTGAGAAGCCATTCACA
>capitella_B_gene_04
TGCAGTGTATGCAATAAAGGATTCAGTCAATCTGGTTATCTGAAAACACACATGAGGATTCACAGTGGTGACA
AGCCATTCGCA
>capitella_B_gene_05
TGCAGTGTATGCAATAAAGGATTCAATCGAGCTGATGTTCTGAAAACACACATGAGGATTCACAGTGGTGACA
AGCCATTCGCA
>capitella_B_gene_06
TGCAGTGTATGCAATAAAGGATTCAATCAAGCTTGTCATCTGAAAACACACATGATGATTCACAGTGGTGACA
AGCCATTCGCA
>capitella_B_gene_07
TGCAGTGTATGCAATAAAGGATTCAATCAATCTGGTGATCTGATAAAACACATGAGGATTCACAGTGGTGAGA
AGCCATTCACA
>capitella_B_gene_08
TGCAGTGTATGCAATAAAGGATTCAGTCAATCTGGTTATCTGAAAACACACATGAGGATTCACAGTGGTGACA
AGCCATTCGCA
>capitella_B_gene_09
TGCAGTGTATGCAATAAAGGATTCAATCGAGCTGATGTTCTGAAAACACACATGAGGATTCACAGTGGTGACA
AGCCATTCGCA
>capitella_C_gene_01
TGCAGTGTATGCAATAAAGGATTTGATCAAGCTGATGTTCTGAAAACACACATGAGGATTCACAGTGGTGAGA
AGCCATTCACA

```
>capitella_C_gene_02
TGCAGTGTATGCAATAAAGGATTCAGTCAATCTGGTGATCTGAAAAAACACATGAGGATTCACAGTGGTGAGA
AGCCATTCGCA
>capitella_C_gene_03
TGCAGTGTATGCAAGAAAGGATTCAGTCAATCTAGTTATCTGAAAACACACATGATGATTCACAGTGGTGAGA
AGCCATTCGCA
>choloepus_hoffmanni__01
TGCAGGGAGTGTGGGCGAGGCTTTGGTCAAAAGCCAAACCTCAGCAGACACCAGAGGACACACACAGGAGAGA
AGCCCTATGTT
>choloepus_hoffmanni__02
TGCAGGGAGTGTGGACGAGGCTTTGGTCGTAAGTCATCACTCATCGTCCACCAGAGGACACACACAGGGGAGA
AGCCCTATGTT
>choloepus_hoffmanni__03
TGCAGGGAGTGTGGACGAGGCTTTGGTCATAAGTCATCACTCATCGTCCACCAGAGGACACACACAGGGGAGA
AGCCCTATGTT
>choloepus_hoffmanni__04
TGCAGGGAGTGTGGACGAGGCTTTGGTCGTAAGTCATCACTCATCGTCCACCAGAGGACACATACAGGGGAGA
AGCCCTATGTT
>choloepus_hoffmanni__05
TGCAGGGAGTGTGGACGAGGCTTTGGTGATAAGTCATCACTCATTTTCCACCAGAGGACACACACAGGGGAGA
AGCCCTATGTT
>choloepus_hoffmanni__06
TGCAGGGAGTGTGGACGAGGCTTTGGTCATAAGTCATCACTCATCGTCCACCAGAGGACACACTCAGGGGAGA
AGCCCTATGTT
>felis_catus__02
TGCAGGGAGTGCGGGCGAGGCTTTACACAGAGGTCAAATCTCTTCAGACACCAGAGGACACACACAGGGGAGA
AGCCCTATGTT
>felis_catus__03
TGCAGGGAGTGCGGGCGAGGCTTTACACAGAGGTCAGATCTCTTCACACACCAGAGGACACACACAGGGGAGA
AGCCCTATGTT
>felis_catus__04
TGCAGGGAGTGCGGGCGAGGCTTCACACGGAGGTCAAATCTCTTCACACACCAGAGGACACACACAGGGGAGA
AGCCCTATGTT
>felis_catus__05
TGCAGGGAGTGTGGGCGAGGCTTTACACGGAGGTCACATCTCTTCACACACCAGAGGACACACACAGGGGAGA
AGCCCTATGTT
>felis_catus__06
TGCAGGGAGTGCGGGCGAGGCTTTACACAGAGGTCAAATCTCTTCACACACCAGAGGACACACACAGGGGAGA
AGCCCTATGTT
>felis_catus__07
TGCAGGGAGTGCGGGCGAGGCTTCACACAGAGGTCAGATCTCTTCAGACACCAGAGGACACACACAGGGGAGA
AGCCCTATGTT
>felis_catus__08
TGCAGGGAGTGTGGGCGAGGCTTTACACAGAGGTCACATCTCTTCACACACCAGAGGACACACACAGGGGAGA
AGCCCTATGTT
>felis_catus__09
TGCAGGGAGTGCGGGCGAGGCTTCACACAGAGGTCAAATCTCTTCAGACACCAGAGGACACACACAGGGGAGA
AGCCCTATGTT
>felis_catus__10
TGCAGGGAGTGTGGGCGAGGCTTTACATGGAGGTCAAATCTCTTCACACACCAGAGGACACACACAGGGGAGA
AGCCCTATGTT
>homo_sapiens__03
TGCAGGGAGTGTGGGCGGGGCTTTAGCTGGAAGTCACACCTCCTCATTCACCAGAGGATACACACAGGGGAGA
AGCCCTATGTC
```

>homo_sapiens__04
TGCAGGGAGTGTGGGCGGGGCTTTAGCTGGCAGTCAGTCCTCCTCACTCACCAGAGGACACACACAGGGGAGA
AGCCCTATGTC
>homo_sapiens__05
TGCAGGGAGTGTGGGCGGGGCTTTAGCCGGCAGTCAGTCCTCCTCACTCACCAGAGGAGACACACAGGGGAGA
AGCCCTATGTC
>homo_sapiens__06
TGCAGGGAGTGTGGGCGGGGCTTTAGCCGGCAGTCAGTCCTCCTCACTCACCAGAGGAGACACACAGGGGAGA
AGCCCTATGTC
>homo_sapiens__07
TGCAGGGAGTGTGGGCGGGGCTTTAGCTGGCAGTCAGTCCTCCTCAGTCACCAGAGGACACACACAGGGGAGA
AGCCCTATGTC
>homo_sapiens__08
TGCAGGGAGTGTGGGCGGGGCTTTAGCTGGCAGTCAGTCCTCCTCACTCACCAGAGGACACACACAGGGGAGA
AGCCCTATGTC
>homo_sapiens__09
TGCAGGGAGTGTGGGCGGGGCTTTAGCAATAAGTCACACCTCCTCAGACACCAGAGGACACACACAGGGGAGA
AGCCCTATGTC
>homo_sapiens__10
TGCAGGGAGTGTGGGCGGGGCTTTCGCGATAAGTCACACCTCCTCAGACACCAGAGGACACACACAGGGGAGA
AGCCCTATGTC
>homo_sapiens__11
TGCAGGGAGTGTGGGCGGGGCTTTAGAGATAAGTCAAACCTCCTCAGTCACCAGAGGACACACACAGGGGAGA
AGCCCTATGTC
>homo_sapiens__12
TGCAGGGAGTGTGGGCGGGGCTTTAGCAATAAGTCACACCTCCTCAGACACCAGAGGACACACACAGGGGAGA
AGCCCTATGTC
>homo_sapiens__13
TGCAGGGAGTGTGGGCGGGGCTTTCGCAATAAGTCACACCTCCTCAGACACCAGAGGACACACACAGGGGAGA
AGCCCTACGTC
>homo_sapiens__14
TGCAGGGAGTGTGGGCGGGGCTTTAGCGATAGGTCAAGCCTCTGCTATCACCAGAGGACACACACAGGGGAGA
AGCCCTACGTC
>lottia_gigantea__01
TGTAATGTTTGTAGTTTTAGTTGTAATCAGGCTTGTAATCTACAGACTCACATGAGAACTCATACTGGAGAGA
AACCCTATAAA
>lottia_gigantea__02
TGTGATGTTTGCAGTTATAGTTGTAAAGTGGCTGGTAGTCTACAGACTCACGTGAGAACTCATACTGGAGAGA
AACCCTATAAA
>lottia_gigantea__03
TGTGATGTTTGTAGTTGTAGTAGTAATCAGGCTGGTCATCTACAGTACCAAATGAGAACCCATACTGGAGAAA
AACCCTATAAA
>lottia_gigantea__04
TGTGATGTTTGTAGTTATAGTTGTAAAGTGGCTGGTAATCTACAGAGACACATGAGAACTCATACTGGAGAGA
AACCCTATAAA
>lottia_gigantea__05
TGTGATGTTTGTAGTTTTAGTAGTAATCAGGCTTGTAATCTACAGACTCACATGAAAACTCATACTGGTGAGA
AACCTTATAAA
>lottia_gigantea__06
TGTGATGTTTGTAGTTTTAGTAGTAATCAGGCTGGTCATCTACAGACTCACATGGGAACTCATACTGGAGAGA
AACCTTATAAA
>lottia_gigantea__07
TGTGATGTTTGTAGTTATAGTTGTAAAGTGGCTGGTCATCTACAGAGACACATGAGAACTCATACTGGAGAAA
AACCCTATAAA

>lottia_gigantea__08
TGTGATGTTTGTAGTTATAGTTGTAAAGTGGCTGGTAATCTACAGAGACACATGAGAACTCATACTGGAGAGA
AACCCTATAAA
>lottia_gigantea__09
TGTGATGTTTGTAGTTATAGTTGTAAAGTGGCTTGTAATCTACAGACTCACATGAAAACTCATACTGGTGAGA
AACCTTATAAA
>lottia_gigantea__10
TGTGATGTTTGTAGTTATAGTTGTAACGTGGCTGGTAGTCTACAGACTCACATGAGAACTCATACTGGAGAAA
AACCTTATAAA
>lottia_gigantea__11
TGTGATGTTTGTAGTTATAGTTGTAATCAGGCTGGTCATCTACAGAGACACATGAGAACTCATACTGGAGAAA
AACCCTATAAA
>lottia_gigantea__12
TGTGATGTTTGTAGTTATAGTTGTAAAGTGGCTGGTAGTCTACAGACTCACATGAGAACTCATACTGGAGAGA
AACCTTATAAA
>lottia_gigantea__13
TGTGATGTTTGTAGTTATAGTTGTAACGTGGCTGGTAGTCTACAGACTCACATGAGAACTCATACTGGAGAAA
AACCCTATAAA
>lottia_gigantea__14
TGTGATGTTTGTAGTTATAGTTGTAATCAGGCTGGTAATCTACAGACTCACATGAGAACTCATACTGGAGAGA
AACCCTATAAA
>lottia_gigantea__15
TGTGATGTTTGTAGTTTTAGTTGTAATCAGGCTGGTAATCTACAGACTCACATGAGAACTCATACTGGAGAGA
AACCTTATAAA
>lottia_gigantea__16
TGTGATGTTTGTAGTTTTAGTTGTAAAGTGGCTGGTAATCTACAGAGTCACATGAGAACTCATACTGGAGAGA
AACCTTATAAA
>lottia_gigantea__17
TGTGATGTTTGTAGTTATAGTTGTAATCGGGCTGGTAGTCTACAGACTCACATGAGAACTCATACTGGAGAGA
AACCTTATAAA
>lottia_gigantea__18
TGTGATGTTTGTAGTTATAGTTGTAATCGGGCTGGTAATCTACAGACTCACATGAGAACTCATACTGGAGAGA
AACCTTATAAA
>lottia_gigantea__19
TGTGATGTTTGTAGTTTTAGTTGTAAAGTGGCTGGTCATCTACAGAGACACATGAGAACTCATACTGGAGAGA
AACCTTATAAA
>lottia_gigantea__20
TGTGATGTTTGTAGTTATAGTTGTAATCAGGCTGGTAGTCTACAGACTCACATGAGAACTCATACTGGAGAGA
AACCTTATAAA
>lottia_gigantea__21
TGTGATGTTTGTAGTTTTAGTTGTAATCAGGCTTGTAATCTACAGACTCACATGAGAACTCATACTGGAGAGA
AACCTTATAAA
>lottia_gigantea__22
TGTGATGTTTGTAGTTTTAGTTGTAATCAGGCTGGTCATCTACAGAGACACATGAAAACTCATACTGGAGAGA
AACCTTATAAA
>lottia_gigantea__23
TGTGATGTTTGTAGTTATAGTTGTAATCGGGCTGGTAGTCTACAGACTCACATGAGAACTCATACTGGAGAGA
AACCCTATAAA
>loxodonta_africana__01
TGTGGAGAGTGTGGACAAGGCTTTAGCCAGAAGTCAGTCCTCATCAGGCACCAGAAGACACACTCGGGGGAGA
AGCCCTATGTC
>loxodonta_africana__02
TGCGGGGAGTGTGGGCGAGGCTTTAGTGTAAAGTCAGTCCTCATCAAACACCAGAGGACACACTCGGGGGAGA
AGCCCTATGTC

>loxodonta_africana__03
TGCGGGGAGTGTGGGCGAGGCTTTAGTGTAAAGTCAGTCCTCATCACACACCAGAGGACACACTCGGGGGAGA
AGCCCTATGTC
>loxodonta_africana__04
TGCGGGGAGTGTGGGCGAGGCTTTAGTGTAAAGTCAGTCCTCATCACACACCAGAGGACACACTCGGGGGAGA
AGCCCTATGTC
>loxodonta_africana__05
TGCGGGGAGTGTGGGCGAGGCTTTAGCCAGAAGTCAGATCTCATCAAACACCAGAGGACACACTCAGGGGAGA
AGCCCTACAGC
>loxodonta_africana__06
TGCAGGGAGTGTGGGCGAGGCTTTAGCCGGAAGTCAGTCCTCATCACACACCAGAGGACACACTCAGGGGAGA
AGCCCTATGTC
>loxodonta_africana__07
TGCGGGGAGTGTGGGCGAGGCTTTAGCCAGAAGTCAAACCTCATCACACACCAGAGGACACACTCAGGGGAGA
AGCCCTATGTC
>loxodonta_africana__08
TGCGGGGAGTGTGGGCGAGGCTTTAGCCGGAAGTCAGTCCTCATCACACACCAGAGGACACACTCAGGGGAGA
AGCCCTATGTC
>loxodonta_africana__09
TGCGGGGAGTGTGGGCGAGGCTTTAGCCAGAAGTCAAACCTCATCACACACCAGAGGACACACTCAGGGGAGA
AGCCCTATGTC
>loxodonta_africana__10
TGCGGTGAGTGTGGGCGAGGCTTTAGCCAGAAGTCAGATCTCATCACACACCAGAGGACACACTCAGGGGAGA
AGCCCTACGTC
>loxodonta_africana__11
TGCAGGGAGTGTGGGCGAGGCTTTAGCCGGAAGTCAAACCTCATCACACACCAGAGGACACACTCAGGGGAGA
AGCCCTATGTC
>macaca_mulatta__03
TGCAGGGAGTGTGGGCGGGGCTTTAGCCAGAAGTCAAGCCTCCGCCGTCACCAGAGGACACACACAGGGGAGA
AGCCCTACCTC
>macaca_mulatta__04
TGCAGGGAGTGTGGGCGGGGCTTTAGAGATAATTCAAGCCTCCGCTATCACCAGAGGACACACACAGGGGAGA
AGCCCTACCTC
>macaca_mulatta__05
TGCAGGGAGTGTGGGCGGGGCTTTAGCAATAATTCAGGGCTCTGCTATCACCAGAGGACACACACGGGGGAGA
AGCCCTACCTC
>macaca_mulatta__06
TGCAGGGAGTGTGGGCGGGGCTTTAGCGATAATTCAAGCCTCCACCGTCACCAGAGGACACACACAGGGGAGA
AGCCCTACCTC
>macaca_mulatta__07
TGCAGGGAGTGTGGGCGGGGCTTTAGCAATAATTCAGGCCTCCGCTATCACCAGAGGACACACACAGGGGAGA
AGCCCTACCTC
>macaca_mulatta__08
TGCAGGGAGTGTGGCCGGGGCTTTAGCAATAATTCAGGCCTCCGCCATCACCAGAGAACACACACAGGGGAGA
AGCCCTACCTC
>macaca_mulatta__09
TGCAGGGAGTGTGGGCGGGGCTTTAGCCAGAAGGCAAACCTCCTTCGTCATCAGAGGACACACACAGGGGAGA
AGCCCTACCTC
>meriones_unguiculatus__01
TGCAGGGAGTGTGGGCGGGGCTTTATGCAGAGGTCAAACCTCATCAGTCACCAGAGGACTCACACAGGGGAGA
AGCCCTATGTG
>meriones_unguiculatus__02
TGCAGGGAGTGTGGGCGGGGCTTTATGCAGAGGTCAAACCTCATCAGTCACCAGAGGACTCACACAGGGGAGA
AGCCCTATGTG

>meriones_unguiculatus__03
TGCAGGGAGTGTGGGCGGGGCTTTACAGTGAAGTCAGTCCTCATCAGTCACCAGAGGACTCACACAGGGGAGA
AGCCCTATGTG
>meriones_unguiculatus__04
TGCAGGGAGTGTGGGCGGGGCTTTACAGTGAAGCCACACCTCATCAGTCACCAGAGGACTCACACAGGGGAGA
AGCCCCACGTG
>meriones_unguiculatus__05
TGCAGGGAGTGTGGGCGGGGCTTTACGCAGAGGTCAAACCTCATCAGGCACCAGAGGACTCACACAGGGGAGA
AGCCCTATGTG
>meriones_unguiculatus__06
TGCAGGGAGTGTGGGCGGGGCTTTACAGTGAAGCCACACCTCATCAGTCACCAGAGGACTCACACAGGGGAGA
AGCCCTATGTG
>meriones_unguiculatus__07
TGCAGGGAGTGTGGGCGGGGCTTTACAGTGAAGCCACACCTCATCAGTCACCAGAGGACTCACACAGGAGAGA
AGCCCTATGTG
>meriones_unguiculatus__08
TGCAGGGAGTGTGGGCGGGGCTTTACAGTGAAGTCAGTCCTCATCAGTCACCAGAGGACTCACACAGGGGAGA
AGCCCTATGTG
>meriones_unguiculatus__09
TGCAGGGAGTGTGGGCGGGGCTTTACAGTGAAGTCAGTCCTCATCAGGCACCAGAGGACTCACACAGGGGAGA
AGCCCTATGTG
>meriones_unguiculatus__10
TGCAGGGAGTGTAGGCGGGGCTTTACGCAGAGGTCAACCCTCATCAGGCACCAGAGGACTCACACAGGGGAGA
AGCCCCACGTG
>microcebus_murinus__03
TGCAGGGAGTGTGGGCGAGGCTTTAGCCAAAAGTCAGACCTCCTCAAACACCAGAGGACACACACAGGGGAGA
AGCCCTATGTC
>microcebus_murinus__04
TGCAGGGAGTGTGGGCGAGGCTTTAGCCAAAAGTCACACCTCCTCAGACACCAGAGGACACACACAGGGGAGA
AGCCCTATGTC
>microcebus_murinus__05
TGCAGGGAGTGTGGGCGAGGCTTTAGCCAAAAGTCAGACCTCCTCATACACCAGAGGACACACACAGGGGAGA
AGCCCTATGTC
>microcebus_murinus__06
TGCAGGGAGTGTGGGCGAGGCTTTAGCTGTAAGTCACACCTCCTCATACACCAGAGGACACACACAGGGGAGA
AGCCCTATGTC
>microcebus_murinus__07
TGCAGGGAGTGTGGGCGAGGCTTTAGCTGTAAGTCAAGCCTCCTCATACACCAGAGGACACACACAGGGGAGA
AGCCCTATGTC
>microtus_agrestis__02
TGCAGGGAGTGTGGGCGGGGCTTTACACGGAAGTCAAACCTCAACGTTCACCAGAGGACACACACAGGGGAGA
AGCCCTATGTT
>microtus_agrestis__03
TGCAGGGAGTGTGGGCGGGGCTTTACACGGAAGGCACTCCTCATCAGTCACCAGAGGACACACACAGGGGAGA
AGCCCTATGTT
>microtus_agrestis__04
TGCAGGGAGTGTGGGCGGGGCTTTACACAGAAGGCACTCCTCATCAGTCACCAGAGGACACACACAGGGGAGA
AGCCCTATGTT
>microtus_agrestis__05
TGCAGGGAGTGTGGGCGGGGCTTTACACAGAAGTCATACCTCATTCTTCACCAGAGGACACACACAGGGGAGA
AGCCCTATGTT
>microtus_agrestis__06
TGCAGGGAGTGTGGGCGGGGCTTTACAGGGAAGTCAAACCTCAACGTTCACCAGAGGACACACACAGGGGAGA
AGCCCTATGTT

>microtus_agrestis__07
TGCAGGGAGTGTGGGCGGGGCTTTACACAGAAGTCATACCTCATTCTTCACCAGAGGACACACACAGGGGAGA
AGCCCTATGTT
>microtus_agrestis__08
TGCAGGGAGTGTGGGCGGGGCTTTACAGGGAAGTCACTCCTCATCAGACACCAGAGGACACACACAGGGGAGA
AGCCCTATGTT
>muntiacus_muntjak_vaginalis__01
TGCGGGGAGTGTGGGCAAAGCTTCCATCACGGGTCAGTCCTCATCAGACACCAGAGGACACACACAGGGGAGA
AACCCTATGTT
>muntiacus_muntjak_vaginalis__02
TGCGGGGAGTGTGGGCGAAGCTTCAGTCAGAAGTCAGTCCTCATCAGACACCAGAGGACACACACAGGGGAGA
AGCCCTATGTT
>muntiacus_muntjak_vaginalis__03
TGTGGGGAGTGTGGGCGAAGCTTCAGTCAGAAGTCAGTCCTCATCAGACACCAGAGGACACACACAGGGGAGA
AGCCCTATGTT
>muntiacus_muntjak_vaginalis__04
TGTGGGGAGTGTGGGCGAAGCTTCAGTCAGAAGGCTCATCTCATCACACACCAAAGGACACACACAGGGGAGA
AGCCCTATGTT
>muntiacus_muntjak_vaginalis__05
TGCGGGGAGTGTGGGCGAAGCTTCAGTCAGAAGACCCATCTCATCTCACACAAGAGGACACACACAGGGGAGA
AGCCCTATGTT
>muntiacus_muntjak_vaginalis__06
TGCGGGGAGTGTGGGCGAAGCTTCTGTCAGAAATCAGCCCTCATCAGACACCAGAGGGCACACACAGGGGAGA
AGCCGTATGTT
>muntiacus_muntjak_vaginalis__07
TGTGGGGAGTGTGGGCGAAGCTTCATTCAGAAGTCAGATTTCATCAGACACCAAAGGACACACACAGGGGAGA
AGCCCTATGTT
>muntiacus_muntjak_vaginalis__08
TGCAGGGAGTGTGGGCAAAGCTACAGCGATAAGACAGTCCTCATCACACACGAGAGGACACACACAGGGGAGA
AGCCCTATGTT
>muntiacus_muntjak_vaginalis__09
TGTGGGGAGTGTGGGCGAAGCTACAGTGATAAGACAGTCCTCATCACACACGAGAGGACACACACAGGGGAGA
AGCCCTATGTT
>muntiacus_muntjak_vaginalis__10
TGTGGGGAGTGTGGGCGAAGCTTCCTTTGGAAGTCAGCCCTCATCAGACACCAGAGGACACACACAGGGGAGA
AGCCGTATGCT
>muntiacus_muntjak_vaginalis__11
TGTGGGGACTGTGGGCGAAGCTTCAATCAGAAGTCAAATTTCATCAGACACCAGAGGACACACACAGGGGAGA
AGCCCTATGTT
>muntiacus_reevesi__01
TGCGGGGAGTGTGGGCGAAGCTTCTGTCAGAAGGCTCATCTCATCACACACCAGAGGACACACACAGGGGAGA
AGCCCTATGTT
>muntiacus_reevesi__02
TGCAGGGAGTGTGGGCAAAGCTTCCGTGATAAGTCAAATCTCATCTCACACAGAAGGACACACACAGGGGAGA
AGCCCTATGTT
>muntiacus_reevesi__03
TGTGGGGAGTGTGGGCAAAGCGTCAGTGATAAATCAAACCTCATCAGACACCAGAGGACACACGCAGGAGAGA
AGCCCTATGTT
>muntiacus_reevesi__04
TGCGGGGAGTGTGGGCGAAGCTTCAATTGGAAGTCCCATCTCATCACCCACCAGAGGATACACACAGGGGAGA
AGCCCTATGCT
>muntiacus_reevesi__05
TGCAGGGAGTGTGGGCAAAGCTTCAGTCAGAAGTCCATCCTCATCACCCACCAGAGGACACACACAGGGGAGA
AGCCCTATGTT

>muntiacus_reevesi__06
TGTGGGGAGTGTGGGCGAAGCTTCAGTCAGAAGTCTCTCCTCATCACCCACCAGAGGACACACACAGGGGAGA
AGCCCTATGTT
>muntiacus_reevesi__07
TGTGGGGAGTGTGGGCGAAGCTTCAGTCAGAAGTCTCTCCTCATCACCCACCAGAGGACACACACAGGGGAGA
AGCCCTATGTT
>muntiacus_reevesi__08
TGTATGGAGTGTGGGCGAAGCTTCAGTCAGAAGACCCATCTCATCACCCACCAGAGGACACACACAGGGGAGA
AGCCCTATGTT
>mus_macedonicus__01
TGCAGGGAGTGTGGGCGGGGCTTTACACAGAAGTCAGACCTCATCAAGCACCAGAGGACACACACAGGGGAGA
AGCCCTATGTT
>mus_macedonicus__02
TGCAGGGAGTGTGGGCGGGGCTTTACAGTGAAGTCAGACCTCATCAAGCACCAGAGGACACACACAGGGGAGA
AGCCCTATGTT
>mus_macedonicus__03
TGCAGGGAGTGTGGGCGGGGCTTTACACAGAAGTCAAACCTCATCCAGCACCAGAGGACACACACAGGGGAGA
AGCCCTATGTT
>mus_macedonicus__04
TGCAGGGAGTGTGGGCGGGGCTTTACACAGAAGTCAGACCTCATCAAGCACCAGAGGACACACACAGGGGAGA
AGCCCTATGTT
>mus_macedonicus__05
TGCAGGGAGTGTGGGCGGGGCTTTACAGTGAAGTCACACCTCACCCAGCACCAGAGGACACACACAGGGGAGA
AGCCCTATGTT
>mus_macedonicus__06
TGCAGGGAGTGTGGGCGGGGCTTTACACAGAAGTCAGACCTCATCAAGCACCAGAGGACACACACAGGGGAGA
AGCCCTATGTT
>mus_macedonicus__07
TGCAGGGAGTGTGGGCGGGGCTTTACAGCGAAGTCACACCTCATCAAGCACCAGAGGACACACACAGGGGAGA
AGCCCTATGTT
>mus_macedonicus__08
TGCAGGGAGTGTGGGCGGGGCTTTACACAGAAGTCAAACCTCATCCAGCACCAGAGGACACACACAGGGGAGA
AGCCCTATGTT
>mus_macedonicus__09
TGCAGGGAGTGTGGGCGGGGCTTTACAGCGAAGTCAAACCTCATCAAGCACCAGAGGACACACACAGGGGAGA
AGCCCTATGTT
>mus_macedonicus__10
TGCAGGGAGTGTGGGCGGGGCTTTACACAGAACTCACACCTCACCCAGCACCAGAGGACACACACAGGGGAGA
AGTCCTATGTT
>mus_musculus__03
TGCAGGGAGTGTGGGCGGGGCTTTACACAGAACTCACACCTCATCCAGCACCAGAGGACACACACAGGGGAGA
AGCCCTATGTT
>mus_musculus__04
TGCAGGGAGTGTGGGCGGGGCTTTACACAGAAGTCAGACCTCATCAAGCACCAGAGGACACACACAGGGGAGA
AGCCCTATGTT
>mus_musculus__05
TGCAGGGAGTGTGGGCGGGGCTTTACACAGAAGTCAGACCTCATCAAGCACCAGAGGACACACACAGGGGAGA
AGCCCTATGTT
>mus_musculus__06
TGCAGGGAGTGTGGGCGGGGCTTTACACAGAAGTCAGTCCTCATCAAGCACCAGAGGACACACACAGGGGAGA
AGCCCTATGTT
>mus_musculus__07
TGCAGGGAGTGTGGGCGGGGCTTTACACAGAAGTCAGTCCTCATCAAGCACCAGAGGACACACACAGGGGAGA
AGCCCTATGTT

```
>mus_musculus__08
TGCAGGGAGTGTGGGCGGGGCTTTACAGCGAAGTCAGTCCTCATCCAGCACCAGAGGACACACACAGGGGAGA
AGCCCTATGTT
>mus_musculus__09
TGCAGGGAGTGTGGGCGGGGCTTTACAGCGAAGTCAAACCTCATCCAGCACCAGAGGACACACACAGGGGAGA
AGCCCTATGTT
>mus_musculus__10
TGCAGGGAGTGTGGGCGGGGCTTTACAGCGAAGTCAGTCCTCATCCAGCACCAGAGGACACACACAGGGGAGA
AGCCCTATGTT
>mus_musculus__11
TGCAGGGAGTGTGGGCGGGGCTTTACAGCGAAGTCAGTCCTCATCCAGCACCAGAGGACACACACAGGGGAGA
AGCCCTATGTT
>mus_musculus__12
TGCAGGGAGTGTGGGCGGGGCTTTACACAGAAGTCAAACCTCATCAAGCACCAGAGGACACACACAGGGGAGA
AGCCCTATGTT
>mus_musculus_castaneus__01
TGCAGGGAGTGTGGGCGGGGCTTTACAGCGAAGTCAAACCTCATCCAGCACCAGAGGACACACACAGGGGAGA
AGCCCTATGTT
>mus_musculus_castaneus__10
TGCAGGGAGTGTGGGCGGGGCTTTACAGCGAAGTCAAACCTCATCCAGCACCAGAGGACACACACAGGGGAGA
AGCCCTATGTT
>mus_musculus_castaneus__11
TGCAGGGAGTGTGGGTGGGGCTTTACACAGAAGTCAAACCTCATCAAGCACCAGAGGACACACACAGGGGAGA
AGCCCTATGTT
>mus_musculus_castaneus__02
TGCAGGGAGTGTGGGCGGGGCTTTACACAGAAGTCAGTCCTCATCCAGCACCAGAGGACACACACAGGGGAGA
AGCCCTATGTT
>mus_musculus_castaneus__03
TGCAGGGAGTGTGGGCGGGGCTTTACACAGAAGTCAGACCTCATCAAGCACCAGAGGACACACACAGGGGAGA
AGCCCTATGTT
>mus_musculus_castaneus__04
TGCAGGGAGTGTGGGCGGGGCTTTACAGCGAGGTCAAACCTCATCCAGCACCAGAGGACACACACAGGGGAGA
AGCCCTATGTT
>mus_musculus_castaneus__05
TGCAGGGAGTGTGGGCGGGGCTTTACACAGAAGTCAGACCTCATCAAGCACCAGAGGACACACACAGGGGAGA
AGCCCTATGTT
>mus_musculus_castaneus__06
TGCAGGGAGTGTGGGCGGGGCTTTACAGCGAAGTCAAACCTCATCCAGCACCAGAGGACACACACAGGGGAGA
AGCCCTATGTT
>mus_musculus_castaneus__07
TGCAGGGAGTGTGGGCGGGGCTTTACAGAGAAGTCAAGCCTCATCAAGCACCAGAGGACACACACAGGGGAGA
AGCCCTATGTT
>mus_musculus_castaneus__08
TGCAGGGAGTGTGGGTGGGGCTTTACAGCGAAGTCAAACCTCATCCAGCACCAGAGGACACACACAGGGGAGA
AGCCCTATGTT
>mus_musculus_castaneus__09
TGCAGGGAGTGTGGGCGGGGCTTTACACAGAAGTCAAGCCTCATCAAGCACCAGAGGACACACACAGGGGAGA
AGCCCTATGTT
>mus_pahari__01
TGCAGGGAGTGTGGGCGGGGCTTTACACAGAAGTCAAACCTCATCACGCACCAGAGGACACACACAGGGGAGA
AGCCCTATGTT
>mus_pahari__02
TGCAGGGAGTGTGGGCGGGGCTTTACAGGGAAGTCACCCCTCATCAGGCACCAGAGGACACACACAGGGGAGA
AGCCCTATGTT
```

>mus_pahari__03
TGCAGGGAGTGTGGGCGGGGCTTTACACAGAAGTCAAACCTCATCACGCACCAGAGGACACACACAGGGGAGA
AGCCCTATGTT
>mus_pahari__04
TGCAGGGAGTGTGGGCGGGGCTTTACAGGGAAGTCACCCCTCATCAGGCACCAGAGGACACACACAGGGGAGA
AGCCCTATGTT
>mus_pahari__05
TGCAGGGAGTGTGGGCGGGGCTTTACACAGAAGTCACACCTCATCAAGCACCAGAGGACACACACAGGGGAGA
AGCCCTATGTT
>mus_pahari__06
TGCAGGGAGTGTGGGCGGGGCTTTACAGAGAAGTCAAACCTCATCAAGCACCAGAGGACACACACAGGGGAGA
AGCCCTATGTT
>mus_pahari__07
TGCAGGGAGTGTGGGCGGGGCTTTACACAGAAGTCACCCCTCATCAGGCACCAGAGGACACACACAGGGGAGA
AGCCCTATGTT
>mus_pahari__08
TGCAGGGAGTGTGGGCGGGGCTTTACACAGAAGTCAAACCTCATCACGCACCAGAGGACACACACAGGGGAGA
AGCCCTGTGTT
>mus_spicilegus__01
TGCAGGGAGTGTGGGCGGGGCTTTACACAGAAGTCAAACCTCATCCAGCACCAGAGGACACACACAGGGGAGA
AGCCCTATGTT
>mus_spicilegus__02
TGCAGGGAGTGTGGGCGGGGCTTTACACAGAAGTCAAACCTCATCCAGCACCAGAGGACACACACAGGGGAGA
AGCCCTATGTT
>mus_spicilegus__03
TGCAGGGAGTGTGGGCGGGGCTTTACAGCGAAGTCAGACCTCATCAAGCACCAGAGGACACACACAGGGGAGA
AGCCCTATGTT
>mus_spicilegus__04
TGCAGGGAGTGTGGGCGGGGCTTTACAGTGAAGTCACACCTCACCCAGCACCAGAGGACACACACAGGGGAGA
AGCCCTATGTT
>mus_spicilegus__05
TGCAGGGAGTGTGGGCGGGGCTTTACACAGAAGTCAGACCTCATCAAGCACCAGAGGACACACACAGGGGAGA
AGCCCTATGTT
>mus_spicilegus__06
TGCAGGGAGTGTGGGCGGGGCTTTACAGCGAAGTCACACCTCACCCAGCACCAGAGGACACACACAGGGGAGA
AGCCCTATGTT
>mus_spicilegus__07
TGCAGGGAGTGTGGGCGGGGCTTTACACAGAAGTCAAACCTCATCCAGCACCAGAGGACACACACAGGGGAGA
AGCCCTATGTT
>mus_spicilegus__08
TGCAGGGAGTGTGGGCGGGGCTTTACAGCGAAGTCAAACCTCATCAAGCACCAGAGGACACACACAGGGGAGA
AGCCCTATGTT
>mus_spicilegus__09
TGCAGGGAGTGTGGGCGGGGCTTTACACAGAACTCACACCTCACCCAGCACCAGAGGACACACACAGGGGAGA
AGTCCTATGTT
>mus_spicilegus__10
TGCAGGGAGTGTGGGTGGGGCTTTAAACAGAAGTCGGACCTCATCCAGCACCAGAGGACACATACAAGAGAGA
AGTAATATATT
>nematostella_vectensis__04
TGCACACAGTGCGGTAAAGCGTTTGCACATGCAAGTAGTCTAACAGAACATATGCGGACACACAGTGGGGAGA
AGCCACATAAG
>nematostella_vectensis__05
TGCACACAGTGCGGTAAAGCGTTTGCACGGGCAAGTGTTCTAACAAGACATATGCGGACACACAGTGGGGAGA
AGCCACATAAG

>nematostella_vectensis__06
TGCACACAGTGCGGTAAAGTGTTTGCACATGCTGGTAATCTAACAACACATATGCGGACACACAGTGGGGAGA
AGCCACATAAG
>nematostella_vectensis__07
TGCACACAGTGCGGTAAAGTGTTTGCACAGGCTGGTATTCTAACAACACATATGCGGACACACAGTGGGGAGA
AGCCACATAAG
>nematostella_vectensis__08
TGCACACAGTGCGGTAAAGCGTTTGCACAGGCTAGTAATCTAACAACACATATGCGGACACACAGTGGGGAGA
AGCCACATAAG
>nematostella_vectensis__09
TGCACACAGTGCGGTAAAGTGTTTGCACATGCTGGTAATCTAACAACACATATGCGGACACACAGTGGGGAGA
AGCCACATAAG
>nematostella_vectensis__10
TGCACACAGTGCGGTAAAGTGTTTGCACAGGCTGGTAATCTAACAACACATATGCGGACACACAGTGGGGAGA
AGCCACATAAG
>nematostella_vectensis__11
TGCACACAGTGCGGTAAAGCGTTTGCACATGCAAGTAATCTAACAAAACATATGCGGATACACAGTGGGGAGA
AGCCATATAAG
>oncorhynchus_mykiss__1
TGCTCTCAGTGTGGGAAGAGTTTCCATCGATCAGGAGACTTAAAGGTGCACCAACGTACTCACACAGGAGAAA
AGCCATATCAC
>oncorhynchus_mykiss__2
TGTTCCCAGTGTGGGAAGAGGTTCAGTGTGTCAGGAAATTTAAAGACACACCAGCGTATTCACACAGGAGAGA
GGCTGTATCCC
>oncorhynchus_mykiss__3
TGCTCTCAGTGTGGGAAGAGTTTCCATCGATCAGGAGACTTAAAGGTGCACCAACGTACTCACACAGGAGAAA
AGCCATATCAC
>oncorhynchus_mykiss__4
TGTTCCCAGTGTGGGAAGAGGTTCAGTGTGTCAGGAAATTTAAAGACACACCAGCGTATTCACACAGGAGAGA
GGCCGTGTGAC
>oryctolagus_cuniculus__01
TGCAGGGAGTGTGGGCGAGGCTTCACAGTGCAGTCAGCCCTCATTACTCACCAGACGACACACACAGGGGAGA
AGCCCTATGCT
>oryctolagus_cuniculus__02
TGCAGGGAGTGTGGGCGAGGCTTCACGCAGAAGTCACACCTCATCAGTCACCAGAGGACACACACAGGGGAGA
AGCCCTATGCT
>oryctolagus_cuniculus__03
TGCAGGGAGTGTGGGCGAGGCTTCACGCAGAAGTCACACCTCATCAGTCACCAGAGGACACACACAGGTGAGA
AGCCCTATGCT
>oryctolagus_cuniculus__04
TGCAGGGAGTGTGGGCAAAGCTTCACAGTGAAGTCACACCTCATCAGTCACCAGAGGACACACACAGGGGAGA
AGCCCTATGCT
>oryctolagus_cuniculus__05
TGCAGGGAGTGTGGGCGAGGCTTCACGCAGAAGTCACACCTCATCAGGCACCAGAGGACACACACAGGGGAGA
AGCCCTATGCT
>oryctolagus_cuniculus__06
TGCAGGGAGTGTGGGCGAGGCTTCACGCGGAAGTCAAACCTCATCAGTCACCAGAGGACACACACAGGGGAGA
AGCCCTATGCT
>oryctolagus_cuniculus__07
TGCAGGGAGTGTGGGCGAGGCTTCACAGTGAAGTCAGCCCTCATTACTCACCAGAGGACACACACAGGGGAGA
AGCCCTATGCT
>oryctolagus_cuniculus__08
TGCAGGGAGTGTGGGCGAGGCTTCACAGTGAAGTCAGCCCTCATTACTCACCAGAGGACACACACAGGGGAGA
AGCCCTATGCT

```
>oryctolagus_cuniculus__09
TGCAGGGAGTGTGGGCGAGGCTTCACGCAGAAGTCAAACCTCATCAGTCACCAGAGGACACACACAGGGGAGA
AGCCCTATGCT
>oryctolagus_cuniculus__10
TGCAGGGAGTGTGGGCAAAGCTTCACGTGGAAGTCAAACCTCATCAGTCACCAGAGGACACACACAGGGGAGA
AGCCCTATGCT
>pan_troglodytes_resequenced_03
TGCAGGGAGTGTGGGCGGGGCTTTAGCTGGAAGTCACACCTCCTCAGTCACCAGAGGACACACACAGGGGAGA
AGCCCTATGTC
>pan_troglodytes_resequenced_04
TGCAGGGAGTGTGGGCGGGGCTTTAGCGTTAAGTCAAGCCTCCTCAGTCACCGGACGACACACACAGGGGAGA
AGCCCTATGTC
>pan_troglodytes_resequenced_05
TGCAGGGAGTGTGGGCGGGGCTTTAGCGTTAAGTCAAGCCTCCTCAGTCACCAGAGGACACACACAGGGGAGA
AGCCCTATGTC
>pan_troglodytes_resequenced_06
TGCAGGGAGTGTGGGCGGGGCTTTAGCCAGCAGTCAAACCTCCTCAGTCACCAGAGGACACACACAGGGGAGA
AGCCCTATGTC
>pan_troglodytes_resequenced_07
TGCAGGGAGTGTGGGCGGGGCTTTAGCGTTAAGTCAAGCCTCCTCAGTCACCAGAGGACACACACAGGGGAGA
AGCCCTATGTC
>pan_troglodytes_resequenced_08
TGCAGGGAGTGTGGGCGGGGCTTTAGCCATAAGTCAAGCCTCCTCAGTCACCAGAGGACACACACAGGGGAGA
AGCCCTATGTC
>pan_troglodytes_resequenced_09
TGCAGGGAGTGTGGGCGGGGCTTTAGCCAGCAGTCACACCTCCTCAGTCACCAGAGGACACACACAGGGGAGA
AGCCCTATGTC
>pan_troglodytes_resequenced_10
TGCAGGGAGTGTGGGCGGGGCTTTAGCAAGCAGTCACACCTCCTCAGTCACCAGAGGACACACACAGGGGAGA
AGCCCTATGTC
>pan_troglodytes_resequenced_11
TGCAGGGAGTGTGGGCGGGGCTTTAGCGTTAAGTCAAGCCTCCTCAGTCACCAGAGGACACACACAGGGGAGA
AGCCCTATGTC
>pan_troglodytes_resequenced_12
TGCAGGGAGTGTGGGCGGGGCTTTAGCCAGCAGTCACACCTCCTCAGACACCAGAGGACACACACAGGGGAGA
AGCCCTATGTC
>pan_troglodytes_resequenced_13
TGCAGGGAGTGTGGGCGGGGCTTTAGCGTTAAGTCAAGCCTCCTCAGTCACCAGAGGACACACACAGGGGAGA
AGCCCTATGTC
>pan_troglodytes_resequenced_14
TGCAGGGAGTGTGGGCGGGGCTTTAGCGTTAAGTCAAGCCTCCTCAGTCACCAGAGGACACACACAGGGGAGA
AGCCCTATGTC
>pan_troglodytes_resequenced_15
TGCAGGGAGTGTGAGCGGGGCTTTAGCCAGCAGTCACACCTCCTCAGACACCAGAGGACACACACAGGGGAGA
AGCCCTATGTC
>peromyscus_leucopus__01
TGCAGGGAGTGTGGGCAGGGCTTTACACGGAAGTCATACCTCATCTGTCACCAGAGGACACACACAGGGGAGA
AGCCCTATGTT
>peromyscus_leucopus__02
TGCAGGGAGTGTGGGCAGGGCTTTATACAGAAGTCAGTCCTCATCAGGCACCAGAGGACACACACAGGGGAGA
AGCCCTATGTT
>peromyscus_leucopus__03
TGCAGGGAGTGTGGGCAGGGCTTTACACGGAAGTCATACCTCATCTGTCACCAGAGGACACACACAGGGGAGA
AGCCCTATGTT
```

>peromyscus_leucopus__04
TGCAGGGAGTGTGGGCAGGGCTTTATACAGAAGTCAGTCCTCATCAGGCACCAGAGGACACACACAGGGGAGA
AGCCCTATGTT
>peromyscus_leucopus__05
TGCAGGGAGTGTGGGCAGGGCTTTACATGGAAGTCAGTCCTCATCTGTCACCAGAGGACACACACAGGGGAGA
AGCCCTATGTT
>peromyscus_leucopus__06
TGCAGGGAGTGTGGGCAGGGCTTTACACGGAAGTCATACCTCATCTGTCACCAGAGGACACACACAGGGGAGA
AGCCCTATGTT
>peromyscus_leucopus__07
TGCAGGGAGTGTGGGCAGGGCTTTACATGGAAGTCACACCTCATCAGGCACCAGAGGACACACACAGGGGAGA
AGCCCTATGTT
>peromyscus_leucopus__08
TGCAGGGAGTGTGGGCAGGGCTTTACACGGAAGTCATACCTCATCTGTCACCAGAGGACACACACAGGGGAGA
AGCCCTATGTT
>peromyscus_leucopus__09
TGCAGGGAGTGTGGGCAGGGCTTTATACAGAAGTCACACCTCATCTGTCACCAGAGGACACACACAGGGGAGA
AGCCCTATGTT
>peromyscus_leucopus__10
TGCAGGGAGTGTGGGCAGGGCTTTACACGGAAGTCATACCTCATCTGTCACCAGAGGACACACACAGGGGAGA
AGCCCTATGTT
>peromyscus_leucopus__11
TGCAGGGAGTGTGGGCAGGGCTTTACATGGAAGTCAGTCCTCATCAGGCACCAGAGGACACACACAGCGGAGA
AGCCCTATCTT
>peromyscus_maniculatus__01
TGCAGGGAGTGTGGGCAGGGCTTTACATGGAAGTCAGTCCTCATCAGGCACCAGAGGACACACACAGGGGAGA
AGCCCTATGTT
>peromyscus_maniculatus__02
TGCAGGGAGTGTGGGCAGGGCTTTACATGGAAGTCAGTCCTCATCTGTCACCAGAGGACACACACAGGGGAGA
AGCCCTATGTT
>peromyscus_maniculatus__03
TGCAGGGAGTGTGGGCAGGGCTTTACATGGAAGTCAGTCCTCATCTGTCACCAGAGGACACACACAGGGGAGA
AGCCCTATGTT
>peromyscus_maniculatus__04
TGCAGGGAGTGTGGGCAGGGCTTTATACAGAAGTCACACCTCATCAGGCACCAGAGGACACACACAGGGGAGA
AGCCCTATGTT
>peromyscus_maniculatus__05
TGCAGGGAGTGTGGGCAGGGCTTTATACGGAAGTCACACCTCATCTGTCACCAGAGGACACACACAGGGGAGA
AGCCCTATGTT
>peromyscus_maniculatus__06
TGCAGGGAGTGTGGGCAGGGCTTTGCACAGAAGTCAGTCCTCATCTATCACCAGAGGACACACACAGGGGAGA
AGCCCTATGTT
>peromyscus_maniculatus__07
TGCAGGGAGTGTGGGCAGGGCTTTACACGGAAGTCACACCTCATCTGTCACCAGAGGACACACACAGGGGAGA
AGCCCTATGTT
>peromyscus_maniculatus__08
TGCAGGGAGTGTGGGCAGGGCTTTGCACAGAAGTCAGTCCTCATCTGTCACCAGAGGACACACACAGGGGAGA
AGCCCTATGTT
>peromyscus_maniculatus__09
TGCAGGGAGTGTGGGCAGGGCTTTACATGGAAGTCAGTCCTCATCTGTCACCAGAGGACACACACAGGGGAGA
AGCCCTATGTT
>peromyscus_maniculatus__10
TGCAGGGAGTGTGGGCAGGGCTTTATACAGAAGTCACACCTCATCAGGCACCAGAGGACACACACAGGGGAGA
AGCCCTATGTT

>peromyscus_maniculatus__11
TGCAGGGAGTGTGGGCAGGGCTTTATACAGAAGTCGCACCTCATCAGGCACCAGAGGACACACACAGGGGAGA
AGCCCTGTCTT
>peromyscus_polionotus__01
TGCAGGGAGTGTGGGCAGGGCTTTATACAGAAGTCAGTCCTCATCTGTCACCAGAGGACACACACAGGGGAGA
AGCCCTATGTT
>peromyscus_polionotus__02
TGCAGGGAGTGTGGGCAGGGCTTTACGTGGAAGTCACACCTCATCAGGCACCAGAGGACACACACAGGGGAGA
AGCCCTATGTT
>peromyscus_polionotus__03
TGCAGGGAGTGTGGGAAGGGCTTTATACGGAAGTCACACCTCATCTGTCACCAGAGGACACACACAGGGGAGA
AGCCCTATGTT
>peromyscus_polionotus__04
TGCAGGGAGTGTGGGCAGGGCTTTATACAGAAGTCACACCTCATCTGTCACCAGAGGACACACACAGGGGAGA
AGCCCTATGTT
>peromyscus_polionotus__05
TGCAGGGAGTGTGGGCAGGGCTTTACACAGAAGTCAGTCCTCATCTGTCACCAGAGGACACACACAGGGGAGA
AGCCCTATGTT
>peromyscus_polionotus__06
TGCAGGGAGTGTGGGCAGGGCTTTATACGGAAGTCATACCTCATCTGTCACCAGAGGACACACACAGGGGAGA
AGCCCTATGTT
>peromyscus_polionotus__07
TGCAGGGAGTGTGGGAAGGGCTTTACATGGAAGTCAGTCCTCATCAGGCACCAGAGGACACACACAGTGGAGA
AGCCCTGTCTT
>pongo_pygmaeus_borneo_03
TGCAGGGAGTGTGGGCGGGGCTTTAGCCGGCAGTCAGTCCTCCTCATTCACCAGAGGACACACACAGGGGAGA
AGCCCTATGTC
>pongo_pygmaeus_borneo_04
TGCAGGGAGTGTGGGCGGGGCTTTAGCCGGCGGTCAGTCCTCCTCATTCACCAGAGGACACACACAGGGGAGA
AGCCCTACGTC
>pongo_pygmaeus_borneo_05
TGCAGGGAGTGTGGGCGGGGCTTTAGCCAGCAGTCAGTCCTCCTCATTCACCAGAGGACACACACAGGGGAGA
AGCCCTATGTC
>pongo_pygmaeus_borneo_06
TGCAGGGAGTGTGGGCGGGGCTTTAGCCGGCGGTCAGTCCTCCTCATTCACCAGAGGACACACACAGGGGAGA
AGCCCTATGTT
>pongo_pygmaeus_borneo_07
TGTAGGGAGTGTGGGCGAGGCTTTAGCTGGAAGTCAGTCCTCCTCAGACACCAGAGGACACACACAGGGGAGA
AGCCCTACGTC
>pongo_pygmaeus_borneo_08
TGCAGGGAGTGTGGGCGGGGCTTTAGCCAGCAGTCAGTCGTCTTCATTCACCAGAGGACACACACAGGGGAGA
AGCCCTACGTC
>pongo_pygmaeus_borneo_09
TGTAGGGAGTGTGGGCGGGGCTTTAGCGGGAAGTCAGTCCTCTTCAGACACCAGAGGACACACACAGGGGAGA
AGCCCTATGTC
>pongo_pygmaeus_borneo_10
TGTAGGGAGTGTGGGCGGGGCTTTAGGGATAAGTCAGGCGTCTGCTATCACCAGAGGACACACACAGGGGAGA
AGCCCTACGTC
>pongo_pygmaeus_sumatran_03
TGCAGGGAGTGTGGGCGGGGCTTTAGCCGGCAGTCAGTCCTCCTCATTCACCAGAGGACACACACAGGGGAGA
AGCCCTATGTC
>pongo_pygmaeus_sumatran_04
TGCAGGGAGTGTGGGCGGGGCTTTAGCCGGCGGTCAGTCCTCCTCATTCACCAGAGGACACACACAGGGGAGA
AGCCCTACGTC

>pongo_pygmaeus_sumatran_05
TGCAGGGAGTGTGGGCGGGGCTTTAGCCAGCAGTCAGTCCTCCTCATTCACCAGAGGACACACACAGGGGAGA
AGCCCTATGTC
>pongo_pygmaeus_sumatran_06
TGCAGGGAGTGTGGGCGGGGCTTTAGCCGGCGGTCAGTCCTCCTCATTCACCAGAGGACACACACAGGGGAGA
AGCCCTATGTT
>pongo_pygmaeus_sumatran_07
TGTAGGGAGTGTGGGCGGGGCTTTAGCTGGAAGTCAGTCCTCCTCAGACACCAGAGGACACACACAGGGGAGA
AGCCCTACGTC
>pongo_pygmaeus_sumatran_08
TGCAGGGAGTGTGGGCGGGGCTTTAGCCAGCAGTCAGTCGTCTTCATTCACCAGAGGACACACACAGGGGAGA
AGCCCTACGTC
>pongo_pygmaeus_sumatran_09
TGTAGGGAGTGTGGGCGGGGCTTTAGCGGGAAGTCAGTCCTCTTCAGACACCAGAGGACACACACAGGGGAGA
AGCCCTATGTC
>pongo_pygmaeus_sumatran_10
TGCAGGGAGTGTGGGCGGGGCTTTAGCGATAAGTCAGGCGTCTGCTATCACCAGAGGACACACACAGGGGAGA
AGCCCTATGTC
>pongo_pygmaeus_sumatran_11
TGCAGGGAGTGTGGGCGGGGCTTTAGCGTTAAGTCAAACCTCCTCAGTCACCAGAGGACACACACAGAGGAGA
AGCTCTACGTC
>rattus_norvegicus__03
TGCAGGGAGTGTGGGCGGGGCTTTTCACAGAAGTCAGACCTCATCAAGCACCAGAGGACACACACAGAGGAGA
AGCCCTACATT
>rattus_norvegicus__04
TGCAGGGAGTGTGGGCGGGGCTTTACACAGAAGTCAGACCTCATCAAGCACCAGAGGACACACACAGAGGAGA
AGCCCTACATT
>rattus_norvegicus__05
TGCAGGGAGTGTGGGCGGGGCTTTACACAGAAGTCAGACCTCATCAAGCACCAGAGGACACACACAGGGGAGA
AGCCCTACATT
>rattus_norvegicus__06
TGCAGGGAGTGTGGGCGGGGCTTTACACAGAAGTCAGACCTCATCAAGCACCAGAGGACACACACAGAGGAGA
AGCCCTACATT
>rattus_norvegicus__07
TGCAGGGAGTGTGGGCGGGGCTTTACACAGAAGTCAAGCCTCATCCGGCACCAGAGGACACACACAGGGGAGA
AGCCCTACATT
>rattus_norvegicus__08
TGCAGGGAGTGTGGGCTGGGCTTTACACAGAAGTCAAACCTCATCCGGCACCTGAGGACACACACAGGGGAGA
AGCCCTACATT
>rattus_norvegicus__09
TGCAGGGAGTGTGGGCTGGGCTTTACACGGAAGTCAAACCTCATCCAGCACCAGAGGACACACACAGGGGAGA
AGCCCTACATT
>rattus_norvegicus__10
TGCAGGGAGTGTGGGCAGGGCCTTACATGGAAGTCAAGCCTCATCCAGCACCAGAGGACACACACAGGGGAGA
AGCCCTACATT
>rhodnius_prolixus__00
TGTACTGAATGTGATTATAGCAGTAATTTATCTAGTGATTTAAAACAACATATGAAAACACATACAGGCAAGA
AGCCATATAAA
>rhodnius_prolixus__01
TGTACAGAATGTGATAGTAGTACCGTGACAAGT---
AATTTAAAACACCATATGAGCAAACATACAGGCGAGAAGCCATATAAA
>rhodnius_prolixus__02
TGTACTGAATGTGATTATAGTTGTACAATGTCCCATTCTTTAAAACTTCATATGAGAACACATACAGGCGAGA
AGCCATATAAA

>rhodnius_prolixus__03
TGTACTGAATGTGATTATAGTTGTACAATGCCCCAATCTTTAAAACACCATATGAGCAAACATACAGGCAAGA
AGCCATATAAA
>rhodnius_prolixus__04
TGTACAGAATGTGATTATAGTGGTACCGTGTCAAGTAATTTAAAACACCATATGAGCAAACATACTGGCAAGA
AGCCATATAAA
>rhodnius_prolixus__05
TGTACTGAATGTGATTATAGTAGTACAATGTCCCAATCTTTAAAACGACATATGAGAACACATTCAGGCGAGA
AGCCATATAAA
>rhodnius_prolixus__06
TGTACAGAATGTGATTATAGTTGTAAAATGACCCAATCTTTAAAACACCATATGAGCAAACATACAGGCGAGA
AGCCATATAAA
>rhodnius_prolixus__07
TGTACTGAATGTAATTATAGTTGTACAATGTCCCAATCTTTAAAACTACATATGAGAACACATACAGGCGAGA
AGCCATATAAA
>rhodnius_prolixus__08
TGTACTGAATGTGATTATAGTTGTACAATGTCCCATTCTTTAAAACACCATATGAGCAAACATACTGGCAAGA
AGCCATATAAA
>rhodnius_prolixus__09
TGTACTGAATGTGATTATAGTTGTACAATGTCCCAATCTTTAAAACAACATATGAGAACACATACAGGCGAGA
AGCCATATAAA
>rhodnius_prolixus__10
TGTACTGAATGTGATTATAGTAGTTCTAGGTTCGATTCTTTAAAACTTCATATGAGAACACATACAGGCGAGA
AGCCATATAAA
>rhodnius_prolixus__11
TGTGTTGAATGCGAATACAGTAGTGCACAGTACATACATTTAAAACTACATATGAGAACACATACAGGCGAGA
AGCCATATAAA
>rhodnius_prolixus__12
TGTACTGAATGTGATTATAGTTGTACAATGTCCCATTCTTTAAAACTCCATATGAGAACACATACAGGCGAGA
AGCCATATAAA
>rhodnius_prolixus__13
TGTACTGAATGTGATTATAGTAGTACAATGTCCCAATCTTTAAAACACCATATGAGAACACATTCAGGCAAGA
AGCCATATAAA
>salmo_salar__1
TGCTCTCAGTGTGGGAAGAGTTTCCGTCGATCAGGAGACTTAAAGGTGCACCAACGTACTCACACAGGAGAGA
GGCCATATCAC
>salmo_salar__2
TGTTCCCAGTGTGGGAAGAGGTTCAGTGTGTCAGGACATTTAAAGACACACCAGCGTACTCACACAGGAGAGA
GGCCATATCAC
>salmo_salar__3
TGTTCCCAGTGTGGGAAGAGTTTCTGTCGATCAGGAGACTTAAAGGTGCACCAACGTACTCACACAGGAGAGA
GGCCATATCAC
>salmo_salar__4
TGTTCCCAGTGTGGGAAGAGGTTCAGTGTGTCACGACATTTAAAGAGACACCAGCATATTCACACGGGAGAGA
GGCCATATCAC
>sorex_araneus__02
TGCAGGGAGTGTGGGCGAGGCTTCAGTCAAAAGTCACATCTCCTCACACACCAGAGGACACACACAGGGGAGA
AGCCCTATGTT
>sorex_araneus__03
TGCAGGGAGTGTGGGCGAGGCTTCACTGATAGGTCAAGTCTCCTCACACACCAGAGGACACACACAGGGGAGA
AGCCCTATGTT
>sorex_araneus__04
TGCAGGGAGTGTGGGCGAGGCTTCAGTCTAAAGTCAAGTCTCCTCAGACACCAGAGGACACACACAGGGGAGA
AGCCCTATGTT

```
>sorex_araneus__05
TGCAGGGAGTGTGGGCGAGGCTTCAGTCTAAAGTCAAGTCTCCTCACACACCAGAGGACACACACAGGGGAGA
AGCCCTATGTT
>sorex_araneus__06
TGCAGGGAGTGTGGGCGAGGCTTCACTGATAGGTCAAGTCTCCTCACACACCAGAGGACACACACAGGGGAGA
AGCCCTATGTT
>sorex_araneus__07
TGCAGGGAGTGTGGGCGAGGCTTCAGTCTAAAGTCAAGTCTCCTCACACACCAGAGGACACACACAGGGGAGA
AGCCCTATGTT
>sorex_araneus__08
TGCAGGGAGTGTGGGCGAGGCTTCAGTCGAAAGTCAAGTCTCCTCAGACACCAGAGGACACACACAGGGGAGA
AGCCCTATGTC
>strongylocentrotus_purpuratus_A_gene_01
TGTGATCAATGTGGTAAAGCGTTCAGTAATGCACATTGTCTTACAACACATAAACGCATCCATACAGGTGAGA
AGCCCTATGTA
>strongylocentrotus_purpuratus_A_gene_02
TGTGATCAATGTGGTAAGGCATTTAATCAAGTGAATGCTCTCACAAGACATGAACGCATCCATACAAGTGAGA
AGCCCTATGTA
>strongylocentrotus_purpuratus_A_gene_03
TGTGATCAATGTGGTAAGGCATTTAATAAAGCTGGTGATGTCAAAAAACATAAACGAATCCACACAGGTGAGA
AGCCCTATGTA
>strongylocentrotus_purpuratus_A_gene_04
TGTGATCAATGTGGTAAGGCATTTAATCGTGAAGGTAATCTCATAGCACATAAACGCATCCATACAGGTGAGA
AGCCCTATATA
>strongylocentrotus_purpuratus_A_gene_05
TGTGATCAGTGTGGTAAGGCATTTAATAATGGGAGTGATCTCACAAAACATAAACGAATCCATACAGGTGAGA
AGCCCTATGTA
>strongylocentrotus_purpuratus_A_gene_06
TGTGATCAATGTGGTAAGGCATTTAATAATGAACATAATCTCACAACCCATAAACGAATCCATACAGGTGAGA
AGCCATATGTA
>strongylocentrotus_purpuratus_A_gene_07
TGTGATCAATGTGGTAAGGCATTTAATCAAGCTGGTGATCTCAAAAAACATAAACGAATCCACACAGGTGAGA
AGCCCTATGTG
>strongylocentrotus_purpuratus_A_gene_08
TGTGATCAATGTGGTAATGCATTTAGTTGTGAACATCATCTCACAACACATAAACGCATCCATACAGGTGAGA
AGCCCTATGTA
>strongylocentrotus_purpuratus_A_gene_09
TGTGATCAATGTGGTAAGGCATTTAGTTGTGAACATCATCTCACAACACATAAACGCATCCATACAGGTGAGA
AGCCCTATGTA
>strongylocentrotus_purpuratus_A_gene_10
TGTGATCAATGTGATAAGGCAGTTAATCAGGTGGGTGATCTCACAAGACATAAACTTATCCATACAGGTGAGA
AGCCCTATGTA
>strongylocentrotus_purpuratus_B_gene_01
TGTGATCAATGTGGTAAAGCATTCAGAGATGCACATTGTCTTACAACACATAAACACATCCATACAGGTGAGA
ATCCCTATGTA
>strongylocentrotus_purpuratus_B_gene_02
TGTGATCAATGTGGTAAGACATTTACTTATGAACATAATCTCACAAGACATAAACGCATCCATACGGGTGAGA
AGCCCTATGTA
>strongylocentrotus_purpuratus_B_gene_03
TGTGATCAATGTGGTAAGGCATTTAAACGTGAAGATGTCCTCACAAGACATAAATGCATCCATACAGGTGAGA
AGCCCTATGTA
>strongylocentrotus_purpuratus_B_gene_04
TGTGATTACTGTGGTAAGGCATTTAATCAAATAAATAATCTCACAACACATAAACGCACCCATACAGGTGAAA
AGCCCTATGTA
```

>strongylocentrotus_purpuratus_B_gene_05
TGTGATCAATGTGGTAAGGCATTTAATCAAGAACCTCATCTCACAACACATAAACGCATTCATACAGGTGAGA
AGCCCTATGTA
>strongylocentrotus_purpuratus_B_gene_06
TGTGATCAATGTGGTAAGGCATTTAATCGTGAAGATGTTCTCACAATACATAAACGCATCCATACAGGTGAGA
AGCCCTATGTA
>strongylocentrotus_purpuratus_B_gene_07
TGTGATCAATGTGGTAAGGCATTTAATCAAGAACCTAGTCTCACAAGACATAAACGCATCCATACAGGTGAGA
AGCCCTATGTA
>strongylocentrotus_purpuratus_B_gene_08
TGTGATCAATGTGGTAAGGCATTTAATCAAGCTGGTGATCTCAAAAAACATAAACGCATCCATACAGGTGAGA
AGCCCTATGTA
>strongylocentrotus_purpuratus_B_gene_09
TGTGATCAATGTGGTAAAGCATTTAATAATATGGGTGATCTCACAAGACATAAACGCATTCATACAGGTGAGA
AGCCCTATGTA
>sus_scrofa__01
TGCAGTGAATGTAGGAAACGCTTCAATAAGAAATCAAGTCTCATTAAACATCAGAGCAGACATATAAGAGAGA
TAGCCTATGGC
>sus_scrofa__02
TGTGGTAAATGTGGCAAAACCTTTCCCCAGAAGTCACAGTTCATTACACATCACAGGACTCATACAGGAGAAA
AACCTTATAAT
>sus_scrofa__03
TGTAGCCAGTGTGGGAAAGCCTTCTCCCAAAAGTCACAGCTGACATCCCATCAGAGGACACATACAGGGGAGA
AACCATATGAA
>sus_scrofa__04
TGTGGTGAATGTGGGAAAGCTTTCTCACGGAAGTCACATCTCATATCACATTGGAGGACGCACACTGGAGAGA
AACCCTATGGA
>sus_scrofa__05
TGCAGTGAGTGTGGGAGGGCCTTTAGTGAAAAGTCAAATCTCATCAATCATCAGAGGATTCATACAGGAGAGA
AACCTTTTGAG
>sus_scrofa__06
TGTAGGGAATGCGGGAAAGCCTTCAGCAGGAAGTCACAGCTTGTCACACATCACAGGACCCACACAGGAACAA
AACCCTATGGA
>sus_scrofa__07
TGTAGTGATTGTAGAAAAGCCTTCTTTGAGAAATCAGAGCTCGTTAGACATCAGACAATTCATACTGGAGAGA
AACCCTATGAA
>sus_scrofa__08
TGCAGTGAGTGTCGGAAAGCCTTCCGAGAGAGGTCCAGTCTCATTAACCACCAGAGAACCCATACAGGAGAGA
AGCCTCATGGG
>sus_scrofa__09
TGCATCCAGTGTGGGAAAGCCTTCTCCCAGAAGTCGCACCTCTTGTCACATCAGATGACACACACAGGAGAGA
AACCCTTTGTG
>sus_scrofa__10
TGCAGTAAGTGTGGAAAAGCCTTCAGTAGGAAATCTCAACTTGTCAGACATCAGAGAACTCATACAGGAGAGA
AACCCTATGAG
>sus_scrofa__11
TGCAATGAATGTGGCAAAGCCTTCAGTGAAAAACTAAGCCTCACTAATCATCAGAGAATCCACACAGGAGAAA
AACCATATGTA
>sus_scrofa__12
TGCAGTGAGTGTGGAAAAGCCTTTTGTCAGAAGTCACATCTCATATCACATCAGAGGACTCATACAGGAGAGA
AACCCTATGAA
>sus_scrofa__13
TGCACGGAATGCGGGAAAGCCTTTGGTGAAAAGTCAAGTCTTGCAACCCACCAGAGAACCCATACGGGAGAAA
AACCTTATGAA

>sus_scrofa__14
TGCAGGGATTGTGAAAAAGCTTTCTCCCAGAAGTCACAGCTCAATACTCACCAGCGAATTCACACAGGAGAAA
AACCTTATGAA
>sus_scrofa__15
TGTGGTATTTGTCAGAAAGCTTTCTTTGAGAAATCAGAACTAATCAGACATCAAAGAACTCATACAGGAGAAA
AACCTTATGAA
>sus_scrofa__16
TGCAGTGAATGTCGGAAAGCCTTCAGGGAGAAGTCAAGTCTCATCAATCATCAGAGAACACATACAGGAGAGA
AACCCTTTGAA
>sus_scrofa__17
TGCAGTGACTGTGGCAAAGCCTTCTCTCGGAAATCACACCTCATACCTCACCAGAGGACTCACACAGGAGAGA
AACCCTATGGA
>sus_scrofa__18
TGCAGTGAGTGTAGGAAGGCCTTCTCTCAGAAGTCACAGCTTGTTAATCATCAGAGAATTCATACAGGAGAGA
AACCATATCAG
