# Supplementary material for: Accelerated Evolution of the Prdm9 Speciation Gene across Diverse Metazoan Taxa
Source: PLoS Genet. 2009 Dec 4;5(12):e1000753. doi: 10.1371/journal.pgen.1000753 (PMC2779102; doi:10.1371/journal.pgen.1000753)
Supplement: Dataset S2 — Genbank accessions for rodent and human sequences (0.04 MB DOC) [file pgen.1000753.s008.doc]

| **Genbank accessions for rodent *Prdm9* terminal zinc finger sequences:** | |
| --- | --- |
| **Mus musculus castaneus** | FJ899852 |
| **Mus specilegus** | FJ899853 |
| **Mus macedonicus** | FJ899854 |
| **Coelomys parahi** | FJ899855 |
| **Apodemus sylvaticus** | FJ899856 |
| **Meriones unguiculatus** | FJ899857 |
| **Peromyscus leucopus** | FJ899858 |
| **Peromyscus maniculatus** | FJ899859 |
| **Peromyscus polionotus** | FJ899860 |
| **Arvicola terrestris** | FJ899861 |
| **Microtus agrestis** | FJ899862 |

| **Genbank accession for chimpanzee (***Pan troglodytes***) *Prdm9* terminal zinc finger sequence:** | |
| --- | --- |
|  | GU166820 |

| **Genbank accessions for PRDM9 sequences of 50 Chinese normal control samples** | | | |
| --- | --- | --- | --- |
| FJ899863 | FJ899864 | FJ899865 | FJ899866 |
| FJ899867 | FJ899868 | FJ899869 | FJ899870 |
| FJ899871 | FJ899872 | FJ899873 | FJ899874 |
| FJ899875 | FJ899876 | FJ899877 | FJ899878 |
| FJ899879 | FJ899880 | FJ899881 | FJ899882 |
| FJ899883 | FJ899884 | FJ899885 | FJ899886 |
| FJ899887 | FJ899888 | FJ899889 | FJ899890 |
| FJ899891 | FJ899892 | FJ899893 | FJ899894 |
| FJ899895 | FJ899896 | FJ899897 | FJ899898 |
| FJ899899 | FJ899900 | FJ899901 | FJ899902 |
| FJ899903 | FJ899904 | FJ899905 | FJ899906 |
| FJ899907 | FJ899908 | FJ899909 | FJ899910 |
| FJ899911 | FJ899912 |  |  |
